# A comprehensive genomic, transcriptomic and proteomic analysis of a hyperosmotic stress sensitive α-proteobacterium

**DOI:** 10.1186/s12866-015-0404-x

**Published:** 2015-03-26

**Authors:** Christian Kohler, Rogério F Lourenço, Jörg Bernhardt, Dirk Albrecht, Julia Schüler, Michael Hecker, Suely L Gomes

**Affiliations:** Departamento de Bioquímica, Instituto de Química, Universidade de São Paulo, Av. Prof. Lineu Prestes 748, 05508-000 São Paulo, SP Brazil; Institut for Microbiology, Ernst-Moritz-Arndt Universität, Greifswald, Germany; Present address: Friedrich Loeffler Institut for Medical Microbiology, Greifswald, Germany

**Keywords:** Osmotic stress adaptation, Compatible solute, Integrative omic analysis, *Caulobacter crescentus*

## Abstract

**Background:**

With the aim of remaining viable, bacteria must deal with changes in environmental conditions, including increases in external osmolarity. While studies concerning bacterial response to this stress condition have focused on soil, marine and enteric species, this report is about *Caulobacter crescentus*, a species inhabiting freshwater oligotrophic habitats.

**Results:**

A genomic analysis reported in this study shows that most of the classical genes known to be involved in intracellular solute accumulation under osmotic adaptation are missing in *C. crescentus*. Consistent with this observation, growth assays revealed a restricted capability of the bacterium to propagate under hyperosmotic stress, and addition of the compatible solute glycine betaine did not improve bacterial resistance. A combination of transcriptomic and proteomic analyses indicated quite similar changes triggered by the presence of either salt or sucrose, including down-regulation of many housekeeping processes and up-regulation of functions related to environmental adaptation. Furthermore, a GC-MS analysis revealed some metabolites at slightly increased levels in stressed cells, but none of them corresponding to well-established compatible solutes.

**Conclusion:**

Despite a clear response to hyperosmotic stress, it seems that the restricted capability of *C. crescentus* to tolerate this unfavorable condition is probably a consequence of the inability to accumulate intracellular solutes. This finding is consistent with the ecology of the bacterium, which inhabits aquatic environments with low nutrient concentration.

**Electronic supplementary material:**

The online version of this article (doi:10.1186/s12866-015-0404-x) contains supplementary material, which is available to authorized users.

## Background

An increase in the extracellular concentration of either ionic or non-ionic solutes is one of the several conditions to which microorganisms must cope with in order to remain viable. High external osmolarity leads to dehydration of the cytoplasm and reduction of the cell turgor [1,2]. The consequent increase in the cytoplasmic ion concentration causes disturbances on several cellular processes, such as central and secondary metabolic pathways, protein metabolism, and cell motility and development. As no active transport mechanism for water is present, microorganisms must employ one of two basic strategies to counteract the deleterious effects of the hyperosmotic stress. In members of the archaeal group Halobacteriaceae as well as in obligately halophilic bacterial anaerobes, all adapted to high-saline environments, potassium ions (K^+^) are accumulated at very high intracellular concentrations [3,4]. This is related to an extensive protein structure adaptation such as an increased proportion of acidic amino acid residues on the surface, attracting K^+^ and water molecules.

Most other microorganisms adapt to high osmolarity by accumulating a restricted range of highly soluble molecules with no net charge at physiological pH, referred to as compatible solutes owing to their compatibility with cellular processes at high internal concentrations [5,6]. In the Gram-negative bacterium *Escherichia coli*, where the hyperosmotic response has been extensively studied, the initial phase of osmoadaptation is characterized by a fast intracellular accumulation of K^+^ and the counter-ion glutamate [7,8]. While K^+^ is rapidly taken up from the environment within minutes upon the osmotic shock, glutamate is mainly synthesized and accumulates at a much slower rate. K^+^ uptake in *E. coli* is performed by the combined action of three specialized transport systems: the P-type ATPase Kdp, which is energized by ATP hydrolysis, and the Trk and Kup systems, that take up K^+^ in symport with a proton [7,8].

It is assumed that K^+^/glutamate act as a second messenger to trigger and coordinate the subsequent osmotic response, characterized by the uptake and/or synthesis of compatible solutes [9,10]. Glycine betaine is the preferred compatible solute for the majority of bacteria. Although *de novo* synthesis of glycine betaine is restricted to some phototrophic bacteria, many microorganisms, including *E. coli*, are able to take up choline from the surrounding environment and convert it in glycine betaine by the Bet system [11]. In addition, glycine betaine released in the bathing medium by either the primary microbial producers or the decaying activity can be transported into the cells by the transmembrane protein ProP and the multi-component binding-protein-dependent transport system ProU. Both transport systems are also involved in the uptake of the compatible solute proline. Despite the widespread capability of synthesizing proline, Gram-negative bacteria accumulate this solute mainly by the transport systems ProP and ProU. Like proline, the compatible solute carnitine plays a role in osmoadaptation, accumulating into the cells mainly by transport from the external environment. Furthermore, endogenous osmoprotection in Gram-negative bacteria is achieved by synthesis of the nonreducing glucose disaccharide trehalose by using three distinct substrates: activated glucose (OstAB system), glucose oligosaccharides (TreYZ) and the reducing disaccharide maltose (TreS) [12-14].

*Caulobacter crescentus* is an oligotrophic free-living α-proteobacterium, normally found in habitats with very low concentrations of organic substances [15,16]. Genome analysis reveals a large number of genes that could enable an adaptation of the bacterium to dilute aquatic conditions [17]. Besides continuous starvation, *C. crescentus* can also experience fluctuations in other environmental parameters, including increases in the external osmotic pressure. To date only a few genes were reported to be involved in *C. crescentus* response to hyperosmotic stress, including the paralogous extracytoplasmic function (ECF) sigma factors σ^T^ and σ^U^ [18-20]. Therefore, the capability of this bacterium to cope with hyperosmotic stress and the molecular systems involved in this response are still poorly characterized. Here, we list *C. crescentus* genes presumably involved in the synthesis or uptake of classical solutes related to osmoadaptation, analyze the sensitivity of cells to high extracellular concentrations of an ionic (sodium chloride) and a non-ionic (sucrose) solute, and describe changes in global gene expression, protein synthesis and metabolites to characterize the response of *C. crescentus* to hyperosmotic stress.

## Results

### *C. crescentus* lacks most of the classical genes involved in hyperosmotic stress

**Figure 1:**
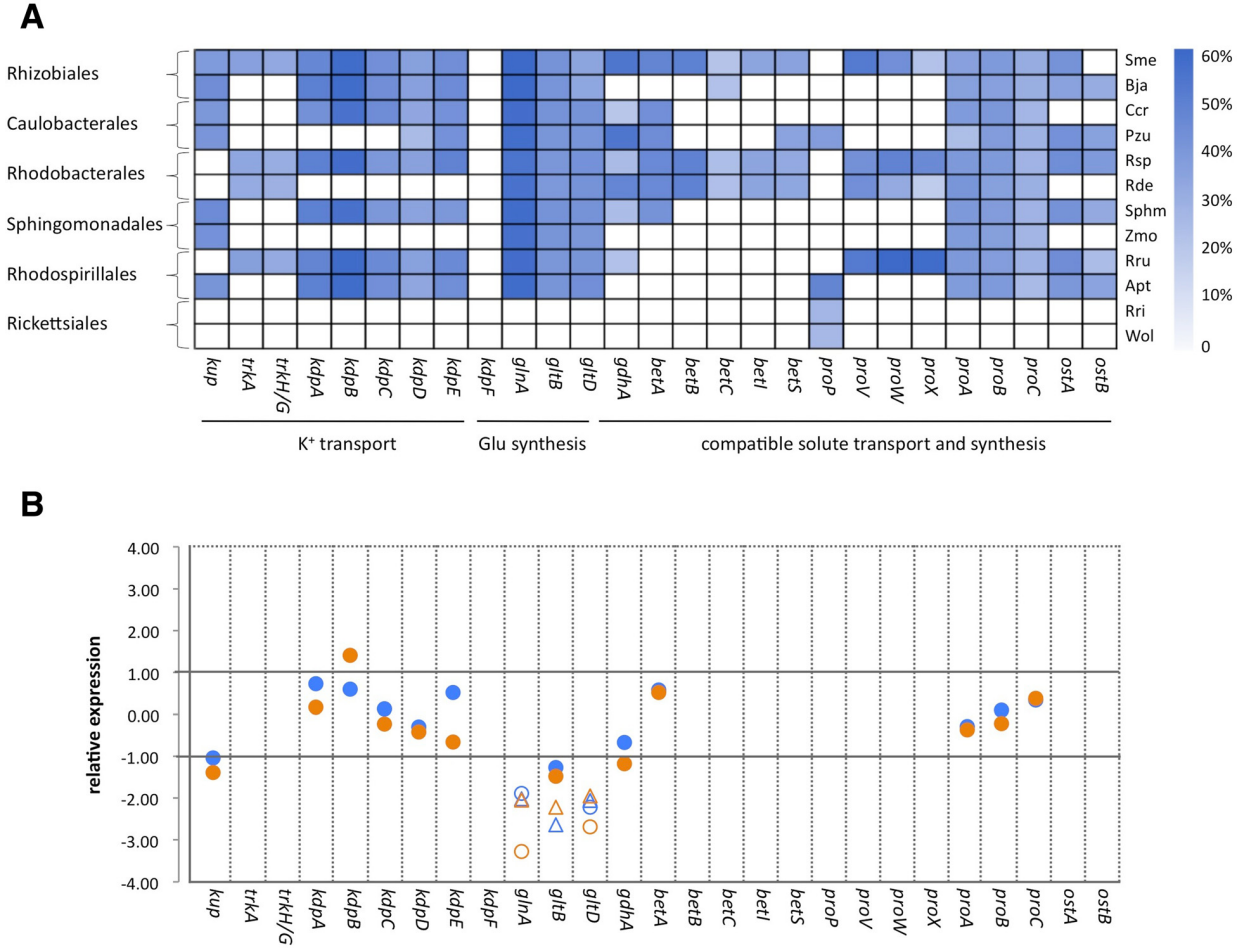
**Potential of** ***C. crescentus*** **in accumulating osmoprotectant solutes. A)** Schematic representation of the repertory of genes putatively related to accumulation of classical solutes under hyperosmotic stress in representatives of each α-proteobacterial family. The intensity of the blue color reflects the identity relative to the corresponding gene of *E. coli* in local sequence alignment; a white box means that the organism does not possess a homolog for the corresponding gene. Sme, Bja, Ccr, Pzu, Rsp, Rde, Sphm, Zmo, Rru, Apt, Rri, Wol and Eco correspond to *Sinorhizobium meliloti* 1021*, Bradyrhizobium japonicum* USDA110*, Caulobacter crescentus* CB15*, Phenylobacterium zucineum*, *Rhodobacter sphaeroides* 2.4.1*, Roseobacter denitrificans*, *Sphingomonas sp*. MM-1, *Zymomonas mobili*s subsp. *mobilis* ZM4, *Rhodospirillum rubrum* ATCC11170*, Acetobacter pasteurianus* IFO 3283–01, *Rickettsia rickettsii* Sheila Smith, *Wolbachia sp*.wMel and *Escherichia coli*. K-12 MG1655, respectively. Accession numbers (locus number) of the corresponding homologs are shown in Additional file 1: Table S1. **B)** Relative abundance of transcript (circle) and protein (triangle) corresponding to genes presumably involved in accumulation of classical solute following either sucrose (blue) or salt stress (orange). Significant changes are denoted as open symbols. Values are log2 and represent the average of three independent biological experiments. Absence of data for a gene indicates that no homologue was identified in *C. crescentus* genome (white color in panel **A**). Particularly for protein data, the absence can also indicate that the corresponding spot was not determined by mass spectrometry analysis, so the relative intensity could not be measured.

As the capability of *C. crescentus* to cope with hyperosmotic stress is not as well addressed as the adaptation to low nutrient environment, we first performed an *in silico* search for genes presumably involved in intracellular accumulation of classical solutes following an increase in the external osmotic pressure. This genomic analysis led to the identification of *C. crescentus* genes belonging to two of the three systems involved in the potassium uptake: Kup (CC_0131) and Kdp (CC_1591 - CC_1595) (Figure 1A and Additional file 1: Table S1). Genes presumably acting on glutamate synthesis were also identified using this approach (CC_1969, CC_2082 and CC_3606 - CC_3607). With respect to compatible solutes, only one gene with a possible role in accumulation of glycine betaine (CC_2642, probably corresponding to the first enzyme in conversion of choline into glycine betaine) and the three genes related to proline synthesis from glutamate (CC_0314, CC_0494 and CC_3430) were found in the genome of *C. crescentus*. Therefore, in theory *C. crescentus* can mediate the uptake of potassium and synthesize glutamate and proline, but has little, if any, ability to accumulate other classical solutes related to osmoadaptation.

With the aim of comparing the expected capability of *C. crescentus* to transport and synthesize classical solutes with that of other α-proteobacteria, we performed similar searches in the genomes of representative species of this bacterial group. Like *C. crescentus*, *Bradyrhizobium japonicum*, *Phenylobacterium zucineum*, *Sphingomonas sp.*, *Zymomonas mobilis*, and *Acetobacter pasteurianus* possess genes related to potassium uptake, but none belonging to the TrK system (Figure 1A and Additional file 1: Table S1). However, genes presumably involved in the synthesis or transport of compatible solutes other than proline were identified in the genome of these α-proteobacterial species, suggesting a more pronounced capacity of these bacteria to undergo the secondary compatible solute based response to increased extracellular osmotic pressure.

The *trK* genes were identified only in *Sinorhizobium meliloti*, *Rhodobacter sphaeroides*, *Roseobacter denitrificans*, and *Rhodospirillum rubrum* (Figure 1A and Additional file 1: Table S1). Curiously, genes related to the uptake and synthesis of compatible solutes were found to be well represented in these α-proteobacteria, suggesting a higher capacity in coping with hyperosmotic stress. Considering the presumable ability to accumulate solute in response to hyperosmotic stress, *Rickettsia rickettsii* and *Wolbachia sp*. were the more compromised of the species analyzed, as these bacteria seem to possess only genes related to proline transport.

Taken together, the *in silico* analysis implies that members of the α-Proteobacteria group are very heterogeneous with respect to the expected capability to transport and synthesize classical solutes following osmotic upshock and that *C. crescentus* is one of the bacteria whose genome is deficient in genes involved in such biological process.

### Sucrose exposure causes a more drastic impact on *C. crescentus* than salt stress

**Figure 2:**
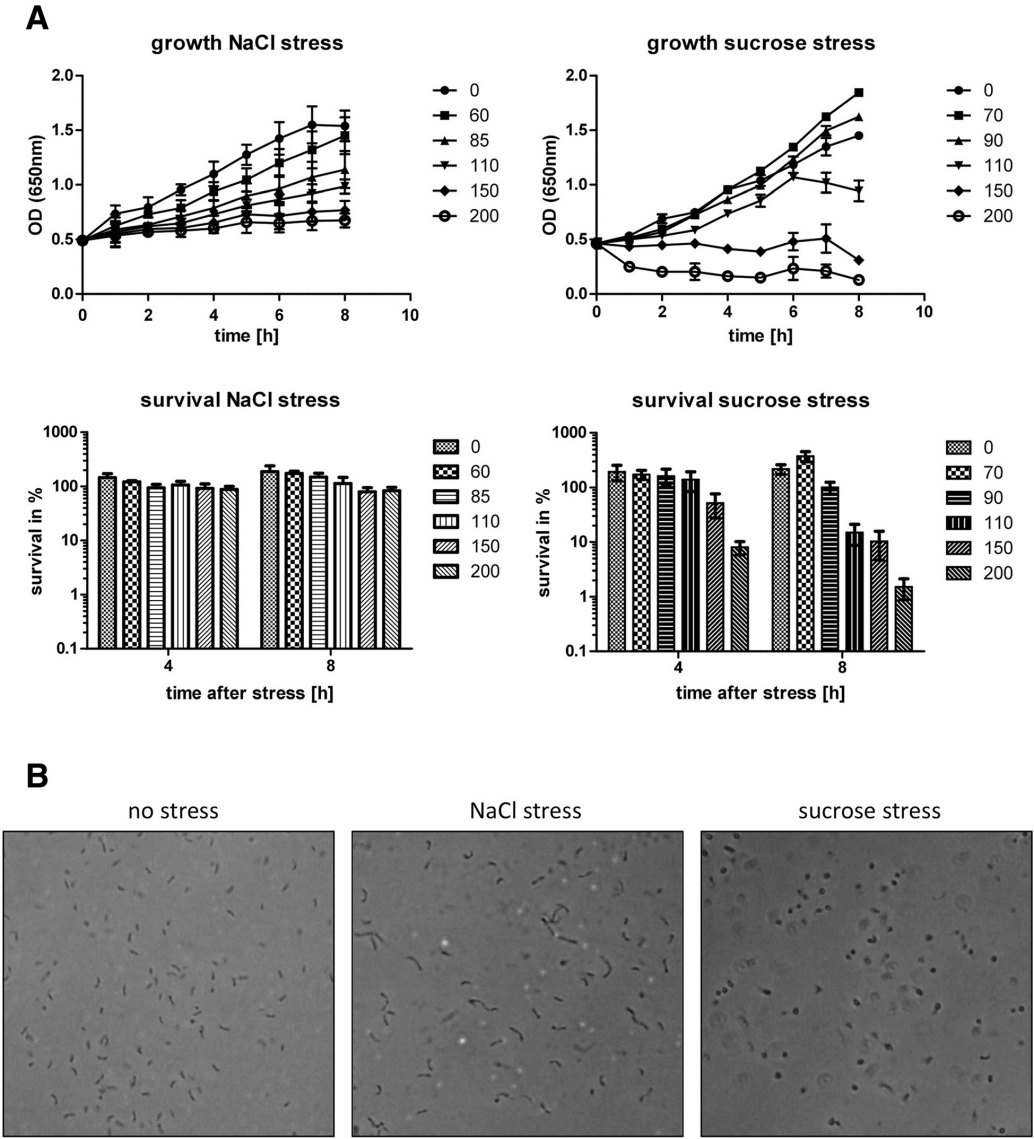
**Ability of** ***C. crescentus*** **to grow and survive under hyperosmotic stress. A)** Growth curves and survival rates of *C. crescentus* in synthetic medium before and after challenge with different concentrations of sodium chloride and sucrose. After reaching an optical density of 0.7 (OD _600 nm_), cells were stressed with sodium chloride or sucrose at concentrations ranging from 40/50 to 200 mM. To measure cell viability, aliquots were removed immediately before and 4h and 8h after exposure to stress condition. Values correspond to the fraction of surviving cells under stress relative to that determined immediately before exposure to stress. Data are mean values of three independent experiments; bars represent the standard error. **B)** Cell morphology under hyperosmotic stress. Exponentially growing cells (OD _600 nm_ = 0.7) were challenged with either 60 mM NaCl or 110 mM sucrose, and phase contrast micrographic images were obtained from cultures kept under these conditions for 24 h. A non-stressed culture was used as control.

When grown in complex medium PYE (peptone yeast extract), *C. crescentus* is known to tolerate up to 85 mM NaCl or 150 mM sucrose [18,20]. Previously, we used the same concentrations to evaluate the involvement of σ^T^ in *C. crescentus* stress response when cultured in minimal salt medium [19,20]. Despite this, the capacity of *C. crescentus* to tolerate hyperosmotic stress in minimal medium, which appears to be more closely related to the condition encountered by the bacterium in its natural habitats, has not been fully addressed. In order to better establish the effect of hyperosmotic stress on *C. crescentus* growth and viability in minimal medium, bacterial cultures propagated to early exponential phase were exposed to different concentrations of either NaCl or sucrose. This analysis showed reduction in the growth rate at NaCl concentrations ranging from 40 to 100 mM (Figure 2A). Surprisingly, bacterial survival was not affected under all concentrations tested, even 8h after more pronounced challenges (treatment with 150 or 200 mM), suggesting that NaCl treatment leads to a decrease in growth rate without resulting in accentuated mortality of the cells.

Sucrose treatment (from 50 to 110 mM) also caused an impact on *C. crescentus* growth in a dose-dependent manner (Figure 2A). However, in contrast to the effect of salt stress, exposure to high sucrose concentrations (110 mM or higher) resulted in decreased viability of the cells. Furthermore, cells at these high sucrose concentrations lost their characteristic morphology throughout the experiments, shifting to a spherical shape (Figure 2B) and becoming more susceptible to lysis after suspension in distilled water. Thus, sucrose stress produces a more pronounced effect upon *C. crescentus*.

Although glycine betaine is known to be a compatible solute that is taken up and/or synthesized in response to osmotic upshift [11], we found no protective effect of this compound upon growth and viability of *C. crescentus* cells exposed to either NaCl or sucrose at concentrations that decreased the rate of growth but did not lead to a complete growth arrest (data not shown). Combined, these results are in accordance to data from *in silico* analysis showing a very restricted or no capability of *C. crescentus* cells in accumulating classical solutes under hyperosmotic stress.

### Global changes in gene expression and protein synthesis

To get a comprehensive picture of the adaptation process to an increase in osmolarity of the medium, we monitored gene expression changes in *C. crescentus* after an osmotic upshift using DNA microarrays. In addition, changes in protein levels of the cytosolic proteome were monitored by two-dimensional gel electrophoresis followed by protein identification by mass spectrometry. As treatment of cells with 110 mM sucrose resulted in a slight growth similar to that observed in the presence of 60 mM NaCl (Figure 2A), and a higher incorporation of L-[^35^S]-methionine into protein was measured under these conditions relative to the complete growth arrested cell cultures, 110 mM sucrose and 60 mM NaCl were chosen for the transcriptomic and proteomic analyses. To minimize secondary effects of the hyperosmotic stress, samples were taken just 30 min after the stress challenge.

By analyzing global transcriptome, 393 genes were identified as differentially expressed following osmotic upshift (Additional file 2: Table S2). In the proteome experiments, 125 protein spots displayed significant change in intensity after stress, and proteins corresponding to 72 spots could be identified (Additional file 3: Figure S1 and Additional file 2: Table S2). About two-thirds of the proteins identified correspond to genes also differentially expressed according to transcriptomic analyses. The remaining proteins were identified only by proteomic analyses, suggesting that both transcriptional and posttranscriptional controls are involved in the regulation of genes under hyperosmotic stress in *C. crescentus*. Together, our experimental procedures allowed us to identify a total of 425 open reading frames whose expression levels changed under at least one of the conditions tested (NaCl or sucrose stress), which are about 10% of all genes predicted in *C. crescentus* genome. Among those open reading frames identified in both experimental approaches as affected by osmotic stress, we could observe a perfect correlation between the transcriptome and the proteome data.

High salinity stress led to up-regulation of 143 genes and resulted in an increased synthesis of 27 proteins, whereas 209 genes and 33 proteins were found to be down-regulated (Additional file 2: Table S2). Sucrose stress increased the expression of 100 genes and 33 proteins and led to down-regulation of 113 genes and 33 proteins. A total of 75 genes and 25 proteins were up-regulated, and 97 genes and 29 proteins were down-regulated under both stress conditions. At a first glance, this seemed to imply that about half of the differentially regulated genes in the presence of NaCl were not regulated after sucrose stress and only few genes were differentially expressed exclusively in the presence of sucrose. However, most of the up- or down-regulated genes observed after high salinity exposure were regulated in the same way after sucrose stress, although many of them did not reach the selected threshold (log_2_ ratio of +1 or −1) (data not shown). Therefore, in general our data suggest that the two different osmotic stressors produce similar changes in gene expression. This is more evident in the protein levels, as just few proteins were differentially synthesized under only one stress condition (Additional file 3: Figure S1 and Additional file 4: Figure S2 and Additional file 2: Table S2).

### Cellular processes affected by osmotic stress in *C. crescentus*

Interestingly, a close inspection in the relative abundance of transcript corresponding to the repertory of genes presumably involved in K^+^ uptake (*kup* and *kdp*ABCDE), glutamate synthesis (*glnA*, *gltAB* and *gdhA*) and proline syntheis (*pro*ABC) showed no significant up-regulation. Instead, *glnA* and *gltD* were down-regulated after both sucrose and salt stresses (Figure 1B). Accordingly, three protein spots whose intensities decreased following hyperosmotic stress corresponded to GlnA, GltB and GltD. Therefore, besides the absence in *C. crescentus* of most of the genes related to classical solutes accumulation, increase in the corresponding transcript and protein under hyperosmotic stress was not ascribed to any of those that are present.

**Figure 3:**
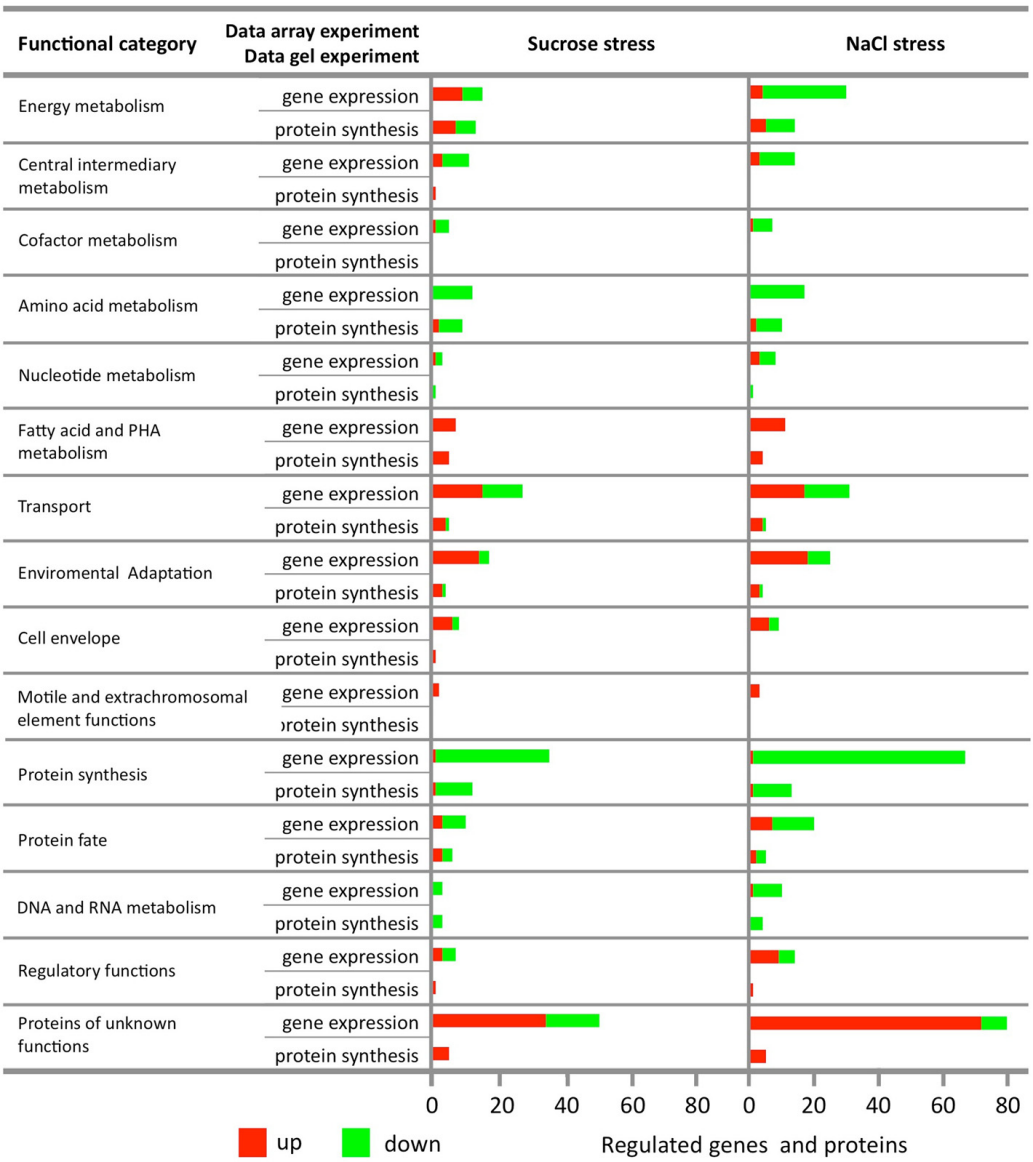
**Global gene expression in** ***C. crescentus*** **under hyperosmotic stress.** Functional classification of up- (red) and down- (green) regulated genes and proteins in *C. crescentus* after challenge to 110 mM sucrose and 60 mM NaCl. Bars indicate the number of genes in each group that were significantly regulated after 30 min of stress.

In order to associate genes with cellular processes, we carried out a thorough reannotation of the additional genes and proteins differentially regulated by searching for conserved domains and sequence similarity. Gene products were then clustered into functional categories (Figure 3). In general, this clusterization indicates that the main functional categories were equivalently enriched under both conditions, further suggesting that, in general, sucrose and NaCl lead to similar responses in the bacterium.

### Protein metabolism

Data showed a general down-regulation of genes/proteins involved in translation, including almost all ribosomal proteins, several translation factors and some proteins required for tRNA synthesis, aminoacylation and ribosome biogenesis. The unique gene/protein acting on translation with increased expression is related to the hibernation factor YfiA, a protein responsible for inactivation of 70S ribosomes by direct binding to the 30S ribosomal subunit [21]. A general decrease in expression was also observed for genes/proteins related to amino acid and folate biosynthesis. Several genes/proteins involved in chaperoning and secretion processes were also down-regulated in the presence of salt or sucrose. Thus, all these changes might suggest a strong reduction in protein synthesis following an increase in the external osmotic pressure.

### Carbon and energy metabolism

We observed down-regulation of several genes/proteins participating in the central metabolism (glycolysis, pyruvate dehydrogenase complex, citric acid cycle, electron transport chain and ATP synthesis). On the other hand, some genes/proteins presumably involved in the Entner Doudoroff pathway were found up-regulated in osmotically stressed cells. Thus, the glucose metabolism seems to be redirected under hyperosmotic stress. Very striking was the observation of the increased expression of genes/proteins involved in the fatty acid metabolism and polyhydroxyalkanoate (PHA) synthesis. This observation points out to the possibility that synthesis of PHA under hyperosmotic stress relies on the fatty acid *de novo* synthesis pathway, as previously shown [22,23]. In addition, genes/proteins involved in the synthesis of polyhydroxybutyrate, a very common kind of PHA, were found up-regulated under the tested stress conditions. Therefore, more than one of the routes for the PHA synthesis might be activated when external osmotic pressure is increased.

### Stress related metabolism and adaptations

Several of the up-regulated genes/proteins have been described previously as stress-responsive genes. In this category are a number of proteins required for degradation of denatured and misfolded proteins and those with a possible detoxification role, such as catalase, glutathione S-transferases, aldehyde dehydrogenases and cytochrome P450 family proteins. Up-regulation of genes/proteins required for detoxification of H_2_O_2_ and toxic organic compounds might suggest that *C. crescentus* suffers oxidative stress under hyperosmotic stress. Curiously, expression of enzymes that detoxify H_2_O_2_ with the expense of reduced coenzyme (glutathione peroxidase and alkyl hydroperoxide reductase) was reduced in the presence of salt or sucrose. Other up-regulated targets include structural porins and enzymes involved in synthesis and degradation of surface sugars. This observation might be indicative of substantial modification in bacterial envelope during the process of adaptation to hyperosmotic stress.

### Transcription and regulatory function

Our analyses revealed a decrease in expression of some genes/proteins related to RNA metabolism, including proteins necessary for RNA unwinding, degradation and synthesis. Interestingly, genes/proteins related to the RNA polymerase core subunits and the principal RNA polymerase sigma factor σ^73^ (RpoD), which is essential for expression of housekeeping genes [24], were down-regulated. Among the regulatory proteins involved in transcription of selected genes under specific conditions, the extracytoplasmic function (ECF) sigma factor σ^T^, σ^U^ and σ^M^, and those involved in the control of expression of the two first sigma factors were found up-regulated [19,20]. In contrast, other regulatory proteins, including the ones involved in phosphate and nitrogen metabolism, were down-regulated. In sum, the changes in expression of these regulatory genes might be responsible for up- and down-regulation of genes following hyperosmotic stress.

### Genes affected specifically or to a greater extent by one of the osmotic stressors

**Figure 4:**
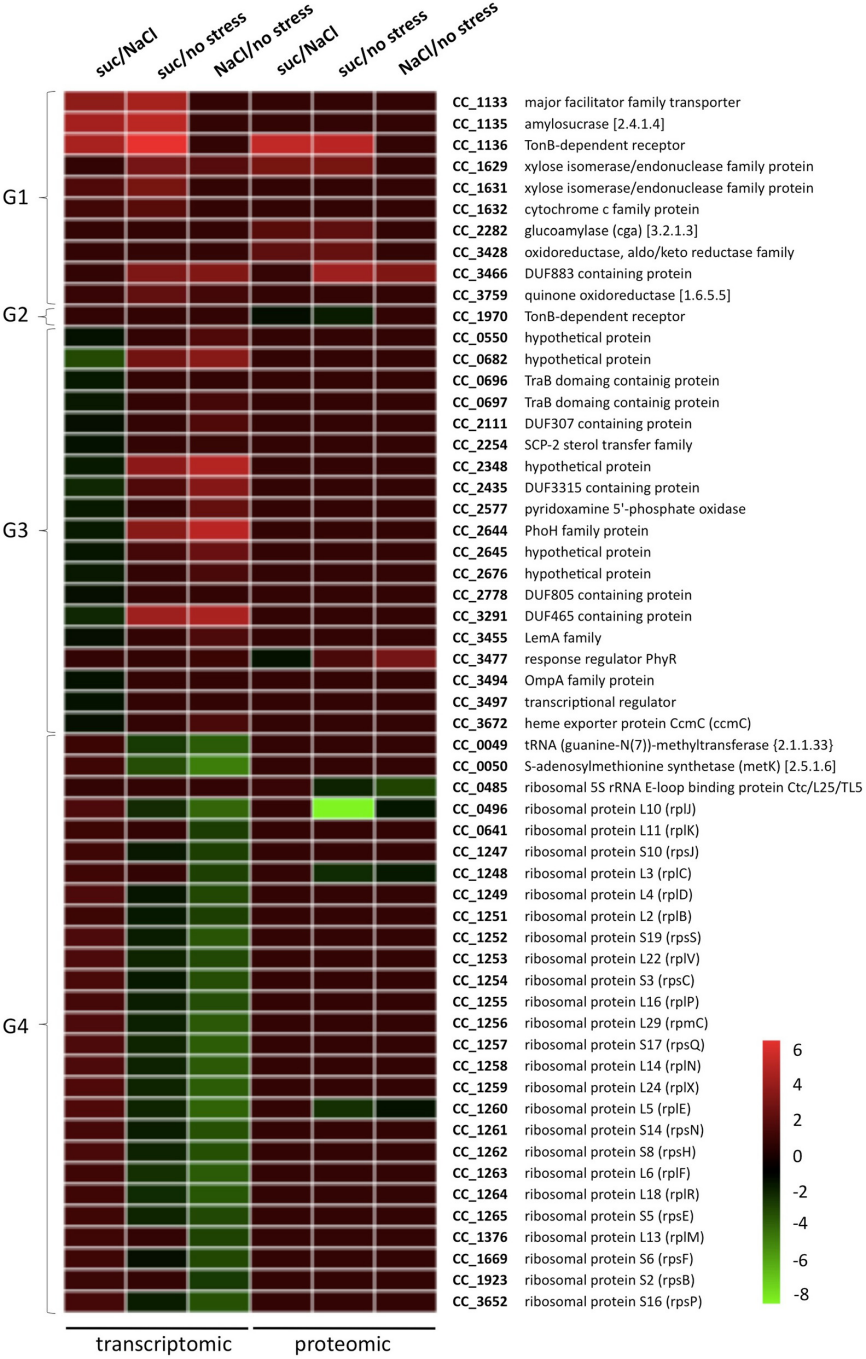
**Dissimilar effects of salt and sucrose upon global gene expression in** ***C. crescentus*** **.** Microarray hybridizations and 2D electrophoresis performed with samples isolated from exponential phase cells immediately before or after exposure to either 110 mM sucrose (suc) or 60 mM NaCl for 30 min. Results shown are the average of three independent biological experiments. Genes and proteins were clustered as following: G1, Higher expression levels in the presence of sucrose or up-regulated only under this stress condition; G2, Down-regulated only under sucrose stress; G3, Higher expression levels in the presence of NaCl or up-regulated only under this stress condition; G4, Lower expression levels in the presence of NaCl or down-regulated only under this stress condition. The complete list of genes and proteins whose expression changed in the presence of either sucrose or NaCl is shown in Additional file 2: Table S2. Gene numbers are according to the CMR (Comprehensive Microbial Resource) annotation.

As suggested above, the two osmotic stressors used in this study seem to produce a similar general change in gene expression and protein synthesis. With the aim of identifying genes and proteins whose expression was specifically or more pronounced changed under one of the osmotic conditions, we conducted additional transcriptomic and proteomic analyses. While the 2-D gels used in this comparison were the same presented above, additional DNA microarray experiments were carried out by directly comparing RNA profile from cells exposed to sucrose with that of NaCl treated cells. In this analysis, transcripts of 32 genes were found at higher levels under sucrose stress and 18 genes showed higher expression during high salinity condition (Figure 4 and Additional file 2: Table S2). At the proteomic level, only 2 proteins were detected at higher levels after NaCl stress and 6 proteins after sucrose stress (Figure 4 and Additional file 3: Figure S1 and Additional file 4: Figure S2 and Additional file 2: Table S2).

By comparing data of this analysis with that obtained when comparisons of transcriptome and proteome were carried out between osmotic shock (NaCl or sucrose exposure) and the non-stress condition, several of the genes/proteins were identified at higher levels under sucrose stress because of their stronger down-regulation following NaCl treatment with respect to sucrose exposure (Figure 4 and Additional file 4: Figure S2). Most of these genes code for ribosomal proteins, suggesting that protein synthesis is more negatively affected under salt stress than following sucrose exposure.

Only a few genes/proteins were affected exclusively or mainly by sucrose stress. A number of these genes are organized in two operons distantly located in the chromosome of *C. crescentus*. One of these operons is CC_1133 - CC_1135, whose genes code for proteins involved in transport across the bacterial envelope and enzymes acting upon carbohydrates. Upstream of this operon is another gene (CC_1136) involved in transport, which was also up-regulated only under sucrose stress (Figure 4). The genes CC_1631 and CC_1632 are part of the operon CC_1628 - CC_1632 and were expressed at higher levels under sucrose stress relative to the salt exposure (Figure 4). Although CC_1628 and CC_1630 were not differentially expressed by comparing transcripts of cells exposed to either sucrose or salt, both genes were up-regulated in the presence of sucrose with respect to the no stress condition. Therefore, all genes belonging to this transcriptional unit are expected to be specially up-regulated under sucrose stress. Genes of this operon also encode proteins with transport or catalytic function.

Many genes expressed at higher levels under salt stress relative to sucrose treatment displayed stronger up-regulation in the presence of NaCl than under sucrose exposure. Most of genes have no putative function assigned yet, and those functionally annotated are predicted to play distinct roles. Therefore, no functional category was enriched with genes expressed at higher levels under high salinity condition. Only one protein (a TonB-dependent receptor) was synthesized at low levels under sucrose stress relative to the salt treatment due to a more pronounced down-regulation after sucrose exposure. Therefore, although the similar general effect of NaCl and sucrose on genes and proteins expression, each stressor leads to small specific changes.

### Analysis of cellular extracts indicates the absence of typical compatible solutes

Results obtained from genomic, transcriptomic and proteomic analyses in this study suggest a severely restricted ability of *C. crescentus* to counteract hyperosmotic stress by synthesizing typical compatible solutes. As described above, CC_1630 is part of an operon up-regulated specifically or mainly in the presence of sucrose and codes for a protein displaying significant similarity to glucose-fructose oxidoreductase (GFOR) from *Z. mobilis* [25,26]. In this bacterium, sorbitol is accumulated in response to an osmotic upshock, being synthesized from the external sugar sucrose by action of the GFOR enzyme. Thus, *Z. mobilis* uses sucrose both as carbon and energy source and to generate sorbitol as an osmoprotective compound. Despite this similarity, a specific search for sorbitol in extracts of cells treated with salt or sucrose by GC-MS analysis revealed that this compatible solute is not produced or its level is under the limit of detection. Therefore, we could exclude a possible role for sorbitol on osmoprotection in *C. crescentus*. In addition, trehalose was not detected in extracts from stressed cells, which fits with our genomic and proteomic data. On the other hand, we found in the extracts slightly higher levels of glutamate, phenylalanine and glucose after hyperosmotic stress trigged by either NaCl or sucrose (Table 1). Besides these compounds, sucrose stressed cells accumulated slightly increased amounts of the amino acids isoleucine, leucine, proline compared to the control.

**Table 1:** **Metabolites significantly changed under hyperosmotic stress in** ***Caulobacter crescentus***

	**Condition** ^**a**^			**t-test** ^**b**^	
**Metabolite**	**Control**	**NaCl stress**	**Sucrose stress**	**NaCl vs control**	**Sucrose vs control**
Glucose	11.85	53.15	64.55	***0.002***	***0.003***
L-glutamate	17.12	78.54	57.92	***<0.001***	***0.001***
L-phenylalanine	0.06	0.14	0.15	***0.008***	***0.020***
L-isoleucine	0.20	0.21	0.59	0.851	***0.010***
L-leucine	0.38	0.64	0.86	0.145	***0.026***
L-proline	0.20	0.36	0.48	0.144	***0.035***

^**a**^Quantification of metabolites according to GC-MS analysis. Values refer to area-substrate peak relative to the area-ribitol peak [20 nM] and are mean of three independent experiments.

^b^Students’ t-test. Values were calculated by comparing relative quantification measured in extract from stressed cells (either 60 mM NaCl or 110 mM sucrose) with that performed in cells growing under non-stress condition. p < 0.05 was considered as statistical significance and values are shown in bold and italic.

In sum, we could show that *C. crescentus* is not able to synthesize the compatible solutes sorbitol and trehalose following treatment with either salt or sucrose, ruling out their involvement as counteracting molecules during hyperosmotic stress. However, a validated protocol for intracellular metabolomic analysis of *C. crescentus* would be needed to clarify all other changes on the metabolome in a deeper detail.

## Discussion

This report describes the capability of *C. crescentus* in tolerating and responding to osmotic upshift triggered by either sugar or salt. Our data showed a very restricted tolerance of this bacterium to hyperosmotic stress, as even a relatively low increase in extracellular concentration of an ionic or non-ionic solute impairs the bacterial propagation in culture. Despite the similar consequence upon growth, salt and sucrose were found to be different with respect to the effect on survival, as cell viability was kept in the presence of high NaCl concentrations, but decreased under more expressive sucrose stress. This indicates that *C. crescentus* is more sensitive to sugar stress than to high salinity condition. This bacterium is well adapted to survive in environments characterized by low concentrations of carbon sources [15,16], where the scarce organic nutrients are scavenged in order to keep essential biological processes and so allow cells retain viability. The low probability of encountering abundant organic nutrients in such environments could have selected for organisms with restricted or no capability to tolerate high concentration of carbohydrates, as the absence of the molecular systems required for the response to these solutes confers the advantage of energy economy related to the maintenance of genetic information and expression of these systems. Accordingly, the distribution of *C. crescentus* in freshwater habitats might be related to the sensitivity to osmotic stress on the whole. Therefore, the ecology of *C. crescentus* suggests that this bacterium does not experience conditions of high external osmolarity in its natural habitats, or this condition is very sporadically encountered.

Data from genomic analysis is in accordance with the low tolerance of *C. crescentus* to high external osmotic pressure, as only some genes related to intracellular accumulation of solutes under this stress condition were identified. As these genes are involved in potassium uptake and synthesis of the amino acids glutamate and proline, their presence could just reflect the requirement of the ion and amino acids for the normal physiology of *C. crescentus*. The down-regulation (glutamate synthesis) or no change (potassium transport and proline synthesis) in expression of genes required for accumulation of osmoprotective solutes observed in transcriptomic and proteomic experiments could favor this hypothesis. The slight increase of glutamate and proline in *C. crescentus* after saline or sucrose stress might be linked with the increased protein degradation during these stresses rather than with activation of biosynthetic pathways. This assumption is supported by i) the up-regulation of various peptidases; ii) the general down-regulation of amino acid biosynthesis, especially those involved in the glutamate synthesis; iii) the increase in the levels of unrelated amino acids (phenylalanine, leucine and isoleucine). Thus, it could be speculated that increased protein degradation is important to supply cells with free amino acids for the biosynthesis of stress related proteins or just means a mechanism to avoid accumulation of misfolded and aggregated proteins. Besides the apparent inability to synthesize glycine betaine, the main compatible solute used by bacteria, *C. crescentus* cells were not beneficiated by the addition of this molecule in the bathing medium, probably because glycine betaine is not transported into the cells. In fact, genes corresponding to uptake systems for this compatible solute (BetT, ProP and ProU) are absent from the genome of *C. crescentus*.

The combined data of transcriptomic, proteomic and metabolomic analyses reported here provide reasons for the striking reduction of *C. crescentus* propagation in the presence of salt or sucrose. Down-regulation of transcripts/proteins related to amino acid biosynthesis, transcription, translation and protein folding suggests that protein synthesis rate is decreased in *C. crescentus* under hyperosmotic stress. In addition, energy conversion seems to be affected as judged by the decreased expression of transcripts/proteins corresponding to metabolic pathways involved in glucose degradation, the carbohydrate used by *C. crescentus* cells during experiments performed in this study. Glucose accumulation in intracellular extracts of stressed cells, as revealed by GC-MS analysis, corroborates this assumption. As protein synthesis and energy conversion are absolutely necessary for bacterial propagation and considering the extension to which they were affected, reduction of both processes might be the main cause of the decrease in growth rate after osmotic upshift. Down-regulation of transcripts/proteins involved in these processes might be caused by decreased expression of the principal sigma factor σ^73^. Conversely, we could rule out the involvement of cell cycle regulator genes in growth arrest during hyperosmotic stress, as no change in the corresponding transcript/proteins was observed.

Whereas an increase in the external osmotic pressure affects essential processes and so reduces bacterial propagation, *C. crescentus* cells are also able to respond to this unfavorable condition by increasing expression of transcripts/proteins related to environmental adaptation. More specifically, these transcripts/proteins could contribute to the modification of the cell envelope and elimination of reactive oxygen species, toxic organic compound and denatured and misfolded proteins that might accumulate in cells under the stress condition. In addition, production of polyhydroxyalkanoate in stressed cells is expected based on the transcriptomic and proteomic data. As the key role of polyhydroxyalkanoate for the hyperosmotic stress resistance in several bacterial species has been reported [27-29], our data could also suggest a possible involvement of accumulation of this class of organic compounds in the *C. crescentus* adaptation following osmotic upshift.

Induction of these functions under hyperosmotic stress appears to be triggered by the proteins supposed or already demonstrated to act in directing RNA polymerase to transcribe specific regulons. One of the up-regulated genes/proteins with this function, the ECF sigma factor σ^T^, was recently reported to be absolutely essential for the osmotic stress response in *C. crescentus*, and 49 genes were identified as belonging to its regulon, including σ^U^ [19,20]. A total of 27 σ^T^-dependent genes were found up-regulated under at least one of the hyperosmotic stress conditions tested here. The use of lower concentration of sucrose in our experiments (110 mM instead of 150 mM) could explain the absence of the other σ^T^-dependent genes. Whereas the function and regulation of σ^T^ and σ^U^ are well described, nothing is known about the role of the ECF sigma factor σ^M^, which was also significantly induced under salt stress condition.

In general, sucrose and NaCl triggered similar responses in *C. crescentus*. Just few genes/proteins were affected specifically or in a greater extent in the presence of one stressor relative to the other. Of particular interest were those transcripts/proteins up-regulated under sucrose stress when compared to cells in the presence of NaCl, as almost all are expected to be involved in transport or metabolism of carbohydrates. Therefore, these genes/proteins could function in carbohydrate utilization. Their induction when the extracellular concentration of carbohydrates increases could allow cells to quickly degrade or store these molecules, which are scarce in the natural environments of the bacterium*.* Alternatively, these genes and proteins could indirectly contribute to the adaptation to high concentrations of this sugar by degrading sucrose, thus causing a decrease in the osmotic pressure of the medium.

Interestingly, most of the genes/proteins affected by osmotic upshift were also differentially expressed upon carbon starvation in *C. crescentus* (see Additional file 2: Table S2 for comparison) [30], including those involved in essential processes such as transcription, translation, energy conversion and transport across membrane, besides a number of proteins of unknown function. Again, changes in expression of the regulatory proteins σ^D^, σ^T^, σ^U^ and σ^M^ appear to be responsible for the effect of carbon starvation upon the physiological functions. The role of σ^T^ and σ^U^ in this response is expected as these sigma factors mediate the general stress response in *C. crescentus*. Instead, the involvement of σ^M^ in hyperosmotic stress and carbon starvation is somewhat surprising, reinforcing the requirement of further characterization of these regulatory genes with the aim of better understanding their contribution for stress adaptation.

Among the free-living α-proteobacteria analyzed with respect to the ability to cope with an increase in the extracellular osmotic pressure, *C. crescentus* is the species with the more reduced set of genes related to accumulation of solute under hyperosmotic stress, and so with the lower expected tolerance to this stress condition. The sensitivity of *C. crescentus* to NaCl is quite similar to that of *B. japonicum*, with both bacteria growing at reduced rate in the presence of about 50 mM NaCl, but not at the concentration of 100 mM [31]. According to our genomic analysis, these bacteria differ only with respect to the occurrence of trehalose related genes, which were found just in *B. japonicum*. The trehalose biosynthetic genes are induced under hyperosmotic stress, allowing accumulation of this compatible solute [32]. However, since we could not detect trehalose by GC-MS analysis, we assume that trehalose does not have an impact as a compatible solute in *C. crescentus*.

Another interesting comparison that deserves attention is with *Z. mobilis*. The genomic analysis shown here in principle suggests a restricted ability to accumulate classical compatible solutes, similar to *C. crescentus*. However, *Z. mobilis* tolerates uncommonly high sucrose concentrations, due to its ability to synthesize and accumulate the unusual compatible solute sorbitol [26]. From our metabolic data, we could exclude sorbitol as a compatible solute for *C. crescentus*. Several characterized α-proteobacterial species, such as *S. meliloti*, *R. sphaeroides* and *R. rubrum* are also very resistant to osmotic upshift, but for these bacteria this feature could be related to their intrinsic capability to accumulate classical compatible solutes by both uptake and synthesis. Finally, in intracellular species such as *R. rickettsii* and *Wolbachia sp.* that are encountered in more controlled environments the complete absence of genes related to accumulation of solutes is justified. In general, the capacity to transport and/or synthesize solutes is correlated with the versatility in occupying natural environments.

Even though the ability to accumulate solutes and the related resistance to hyperosmotic stress is very different among α-proteobacteria, an increase in the external osmotic pressure triggers changes in global gene expression of *C. crescentus* quite similar to those observed for other representatives of this phylogenetic group, including down-regulation of genes involved in protein synthesis and energy metabolism and up-regulation of genes acting upon cell envelope and detoxification functions [33-36]. All these changes are related to a stress condition, in which transcription and translation become reduced and more specific in order to favor physiological modifications required under the harmful condition. Therefore, the striking variation in the capability of α-proteobacteria in tolerating osmotic stress is mainly linked with their ability to accumulate solute.

## Conclusion

Integrative data presented in this study contribute to expand our knowledge about the capacity of *C. crescentus* to tolerate an increase in the external osmotic pressure, as well as the strategies employed by this bacterium to respond to this stress condition. It was possible to correlate the low genomic representation of genes required for intracellular accumulation of solutes with the restricted capability of *C. crescentus* cells to propagate following an osmotic upshift. This correlation prompted us to suppose that the bacterium does not experience hyperosmotic stress in its natural environment or that the increase in external osmotic pressure is very weak, allowing cells to respond to this stress condition and keep viable. Results are therefore consistent with the fact that *C. crescentus* is very well adapted to aquatic habitats containing low amount of inorganic ions and organic compounds.

## Methods

### Bacterial strains and growth conditions

*C. crescentus* strain NA1000 was cultured at 30°C in M2 minimal salts medium plus glucose [37]. Overnight culture of strain NA1000 was used to inoculate fresh M2 minimal salts medium and culture was grown to the exponential growth phase (OD_600_ = 0.7). For osmotic/salt stress experiments, cells were incubated in the presence of sodium chloride (NaCl) or sucrose at final concentrations ranging from 40/50 to 200 mM. Growth curves were constructed by incubating cultures for additional 4 hours. To measure cell viability, aliquots were taken, serially diluted and plated 4 and 8 hours after stress on M2 minimal medium for counting colony-forming units.

### RNA extraction

For isolation of RNA used in DNA microarray experiments, cultures of *C. crescentus* NA1000 were grown to exponential growth phase (OD_600_ = 0.7) and submitted for 30 minutes to stress (60 mM NaCl or 110 mM sucrose) or kept under control conditions (no stress). Cells (four aliquots of 2 ml from each treatment) were collected by centrifugation in a microcentrifuge for 1 min and suspended in 1 ml of Trizol Reagent (Invitrogen). After the extraction procedure according to manufacturer’s instructions, the integrity of the RNA was checked by agarose gel electrophoresis and samples were tested for the absence of DNA contamination by PCR.

### Microarray analysis

Three distinct biological RNA samples isolated from control cells and from each stress condition were reverse transcribed and labeled by using the FairPlay III Microarray Labeling system (Agilent) as previously described [19,38]. Briefly, cDNA was synthesized from total RNA (20 μg) in the presence of amino allyl modified dUTP and random primer. After purification, the resulting amino-modified cDNA was chemically labeled by incorporation of the dyes Alexa Fluor 555 (Cy3) or Alexa Fluor 647 (Cy5). Labeled cDNAs were combined, mixed with Agilent hybridization buffer, and competitively hybridized to custom-designed Agilent microarrays according to the manufacturer’s instructions (Agilent). Data extraction and normalization was performed using Agilent Feature Extraction Software 9.5.3.1 (Agilent). The custom-designed arrays contain 9–11 probes covering a region around predicted translational start sites (−300 to +200 relative to the translational start site +1) of each gene. Only those probes downstream of the translational start site were considered for estimating the fold change of gene expression. Ratios obtained for probes corresponding to the same gene were averaged and genes showing a ratio log2 (stress - NaCl or sucrose/control) < −1 or log2 (stress - NaCl or sucrose /control) > 1 in all three biological replicates were considered as differentially expressed. Microarray datasets were deposited in GEO (GSE49654).

### 2-DE and image generation

Two distinct biological protein samples from each stress and control conditions were isolated. For protein analyses, cultures of *C. crescentus* NA1000 were grown to the exponential growth phase (OD_600_ = 0.7) and submitted for 30 minutes to stress (60 mM NaCl or 110 mM sucrose) or kept under control conditions. Afterwards, pulse-labeling was performed for 5 min by using 25 μCi of L-[^35^S]-methionine per mL. Then, 5 min later, L-[^35^S]-methionine incorporation was stopped by adding an excess of unlabeled methionine (1 mM) and chloramphenicol (100 mg/mL) and subsequently by transferring the sample to ice. After disruption of the harvested cells by sonication, the protein solution was separated from cell debris by centrifugation, and the protein amount and incorporated radioactivity were determined. Crude protein extracts (100 μg of protein) were loaded onto Pharmacia ready-made IPG strips (pH range 3–10) for the IEF of 2-DE as recommended. The separation in the second dimension was carried out as suggested by the manufacturer. After fixing with ethanol, acetic acid and water (40/10/50% vol/vol), the wet gels were dried on a chromatography paper backing by using a heated vacuum dryer. For autoradiography of the incorporated ^35^S methionine, dried gels were exposed to storage phosphor screens (Molecular Dynamics Storage Phosphor Screen, 20 by 25 cm) for a time span ensuring usage of the whole dynamic range by the strongest spot and corresponding to the amount of radioactivity separated on the gel. Screens were scanned using a Typhoon 9400 Variable Mode Imager (Amersham Biosciences) at 65 536 (16 bit) graylevels) and a resolution of 200 micron.

### 2-D gel analysis

To visualize changes in the protein pattern we used dual channel imaging as supported by Delta2D 4.3 (DECODON Greifswald). This technique involves overlaying the protein synthesis images of the control (pseudocolored green) and the protein synthesis image of the stress- NaCl or sucrose (pseudocolored red) onto each other [39]. To ensure that results were not influenced by spot mismatches, distorted gels were adjusted by using the program DECODON Delta2D 4.3 [40]. After pseudocolor overlaying by DECODON Delta2D, the histograms of the autoradiograms were normalized according their gray level integrated overall quantity. This procedure generates a dual channel image with an equal representation of detectable quantities of each sub-image. For spot detection on autoradiograms the images were fused using the union image fusion algorithm of Delta2D. After image fusion, spot positions and boundaries were detected on the fused image (proteome map). Their shapes and positions were transferred to each single autoradiogram and were subsequently quantified by using grey level integration inside spot boundaries and normalized by using 100% detected overall gel quantity as reference. An RSD of 30–40% is considered as sufficient in parallels of 2-D gel experiments. The reproducibility of our data was high, with more than 90% of all data of the identified proteins falling below an RSD of 35%.

For the extraction of differentially expressed spots, relative spot volumes were standardized (mean centering and division by standard deviation). The significance of changes in the protein synthesis of detected spots was determined by one-factorial ANOVA for each spot considering both the stress types (α = 0.1, P-values based on F-distribution) supported by Mev 4.1 [41]. Low abundant spots with a spot volume less than 0.02% on all images were eliminated from these analyses. Finally, changes of the average values exceeding two-fold up- or down-regulation were considered as substantial.

### Protein identification

For identification of proteins by mass spectrometry, preparative 2D PAGE was performed. Some 300 μg of protein extracts was loaded onto IPG strips (GE-Healthcare) in the pH range of 3–10. The resulting 2D gels were fixed in the presence of Sypro Ruby. The stained gels were scanned with the Typhoon 9400 Variable Mode Imager (Amersham Biosciences). For identification of proteins by MALDI-TOF-MS, Sypro Ruby stained protein spots were cut out from gels using a spot cutter (Proteome WorkTM) with a picker head of 2 mm and transferred into 96-well microtiter plates. Digestion with trypsin and subsequent spotting of peptide solutions onto the MALDI targets were performed automatically in the Ettan Spot Handling Workstation (GE-Healthcare) using a modified standard protocol [42]. MALDI-TOF-MS analyses of spotted peptide solutions were carried out on a Proteome-Analyzer 4700 (Applied Biosystems, Foster City, CA, USA). The spectra were recorded in reflector mode in a mass range from 900 to 3700 Da. Automatic or manual calibration was performed as described previously [42]. After calibration, peak lists were created using the ‘peak to mascot’ script of the 4700 ExplorerTM software. The resulting peak lists were analyzed by using the mascot search engine (Matrix Science, London, UK), GPMAW 4.1 (Lighthouse data). The annotation of *C. crescentus* CB15 was used for protein identification and denotation. Peptide mixtures that yielded at least twice a Mowes score of at least 50 and sequence coverage of at least 30% was regarded as positive identifications. Proteins that failed to exceed the 30% sequence coverage cut-off were subjected to MALDI-MS/MS [42].

### Integrated transcriptome and proteome data representation

Treemaps of gene functional categories of *C. crescentus* have been constructed according to the TIGRFam classification [43]. For this purpose the 2D plane was iteratively partitioned according to the TIGR main roles, sub-roles, *C. crescentus* predicted operons and proteins [44]. The treemap cells on the deepest hierarchy level represent the *C. crescentus* proteins. Treemap cells were reordered to ensure that consecutive genes in the genome are symbolized by consecutive cells. Circular nodes represent the genes, the connectors in between link operon members. Gene expression was encoded by using a color ramp starting at blue (decreased expression) via grey (no change) and ending at orange (high expression). Messenger RNA data were used for coloring the gene circles and connectors, protein data for coloring the treemap cells. Dark grey cells have no assigned data.

### Cell sampling for GC-MS analysis

*C. crescentus* were cultivated in M2 to an OD_600_ of 0.5 and stressed with either 60 mM NaCl or 110 mM sucrose. After 3 hours, equal amount of control and stressed cells were harvested by centrifugation at 12000 × g for 3 minutes at 2°C. Supernatants were discarded and the cell pellets were overlayed with 75% (w/v) ethanol −20°C. The tubes were imminently frozen in liquid nitrogen until the final sample preparation.

### Sample preparation for GC-MS analysis

All samples were thawed on ice and the cell pellets were disrupted using a Ribolyser (MagNa Lyser, Roche, Germany), at 5000 m × s for 30 seconds. Samples were transferred on 50 ml falcon tubes and the ribolyser tubes were washed with a total of 5 ml 75% (w/v) ethanol −20°C until all cell rests are washed out and were transferred to the falcon tube. Next, the internal standards were added (20 nmol ribitol, 20 nmol norvaline and 2.5 nmol camphorsulfonic acid; Sigma-Aldrich) and the extraction solution was mixed and incubated for 10 min on ice. Afterwards, the Falcon tubes were filled with destilated water to the final volume of 35 ml and stored at – 70°C. Finally, the samples were lyophilized for GC-MS analysis.

### GC-MS analysis

Lyophilized samples were derivatized using a two-step method with MeOX (Sigma-Aldrich) and MSTFA (Chromatographie Service GmbH) as described [45]. For identification and quantification of metabolites a GC-MS method was applied as described previously [46]. Qualitative and quantitative analysis were performed using the ChromaTOF software (LECO Corporation). Identification of peaks was carried out by comparison of mass-spectra and retention time with an in-house database and the Fiehn GC/MS metabolomics RTL library (Agilent Technologies).

### *In silico* search for genes related to accumulation of typical osmoprotective solutes

The Kyoto Encyclopedia of Genes and Genomes (KEGG) database was used in a search for the annotated open reading frame displaying the highest identity to each gene known in *E. coli* to be involved in potassium transport (*kup*, *trkAHG* and *kdpABCDEF*), glutamate synthesis (*glnA*, *gltBD* and *gdhA*), glycine betaine synthesis (*betABCI*), glycine betaine transport (*betS*), glycine betaine, proline and carnitine transport (*proP* and *proVWZ*) proline synthesis (*proABC*) and trehalose synthesis (*ostAB*). This analysis resulted in an initial list of genes in representative α-proteobacterial species (*Sinorhizobium meliloti* 1021, *Bradyrhizobium japonicum* USDA110, *Caulobacter crescentus* CB15, *Phenylobacterium zucineum*, *Rhodobacter sphaeroides* 2.4.1, *Roseobacter denitrificans*, *Sphingomonas sp.* MM-1, *Zymomonas mobilis* subsp. *mobilis* ZM4, *Rhodospirillum rubrum* ATCC11170, *Acetobacter pasteurianus* IFO3283-01 *Rickettsia rickettsii* Sheila Smith and *Wolbachia sp.* wMel). Each gene from this list was used in a similar search for the annotated open reading frame displaying the highest identity in *E. coli* and the other α-proteobacterial species. In both searches, the threshold for Smith-Waterman score was 100 and the minimal overlap in local sequence alignment considered was 75% of the hit’s length. Genes identified in this best hit reciprocal search were assumed to be present in the α-proteobacteria analyzed. All genes uncovered by this *in silico* analysis are shown in Additional file 1: Table S1. Protein domains were ascribed according to the Pfam protein families database [47].

## Additional files

Additional file 1: Table S1. Distribution of genes related to classical solute accumulation under hyperosmotic stress in α-proteobacterial representatives. Additional file 2: Table S2. Genes and proteins differentially expressed under hyperosmotic stress in *Caulobacter* crescentus. Additional file 3: Figure S1. Proteomic analysis of stresses cells. Panels display false colored dual channel images of the synthesis of proteins after 30 min as revealed by radioactive labeling for 5 min. All significantly regulated proteins are labeled in their appropriate color (green or red). Delta 2D software was used to visualize complex protein expression patterns on the 2D image in the standard pH range of 3 to 10. Additional file 4: Figure S2. TIGRFam Treemaps of regulated *C. crescentus* genes (circles) and proteins (cells) after osmotic stress found by global transcriptomic and proteomic analyses. (A) Treemap for the response to sucrose stress, (B) Treemap for the response to NaCl stress and (C) Treemap showing the comparison between sucrose and NaCl stress. Figures on left side show the results on the level of functional category/subcategory and on the right side on the level of genes and proteins. Blue colors show repressed genes and orange colors the induction of genes. The intensity of the colors reflects the strength of repression or induction.
