# Supplementary material for: A comprehensive genomic, transcriptomic and proteomic analysis of a hyperosmotic stress sensitive α-proteobacterium
Source: BMC Microbiol. 2015 Mar 26;15:71. doi: 10.1186/s12866-015-0404-x (PMC4391529; doi:10.1186/s12866-015-0404-x)
Supplement: Additional file 1: Table S1. — Distribution of genes related to classical solute accumulation under hyperosmotic stress in α-proteobacterial representatives. [file 12866_2015_404_MOESM1_ESM.pdf]

Table S1. Distribution of genes related to classical solute accumulation under hyperosmotic stress in α-proteobacterial representatives

Potassium transport system

kup

Sme	Bja	Ccr	Pzu	Rsp	Rde	Sphm	Zmo	Rru	Apt	Rri	Wol	Eco	Pfam motif
SMc00873	blr3803	CC_0131	PHZ_c0050	NI	NI	G432_00850	ZMO1209	NI	APA01_23240	NI	NI	b3747	K_trans

trkA

Sme	Bja	Ccr	Pzu	Rsp	Rde	Sphm	Zmo	Rru	Apt	Rri	Wol	Eco	Pfam motif
SMc01046	NI	NI	NI	RSP_2841	RD1_2757	NI	NI	Rru_A1681	NI	NI	NI	b3290	TrkA_N, TrkA_C

trkH/G

Sme	Bja	Ccr	Pzu	Rsp	Rde	Sphm	Zmo	Rru	Apt	Rri	Wol	Eco	Pfam motif
SMc00937	NI	NI	NI	RPS_1854	RD1_1942	NI	NI	Rru_A1134	NI	NI	NI	b1363, b3849	TrkH

kdpA

Sme	Bja	Ccr	Pzu	Rsp	Rde	Sphm	Zmo	Rru	Apt	Rri	Wol	Eco	Pfam motif
SMa2333	bll6779	CC_1591	NI	RSP_1265	NI	G432_12245	NI	Rru_A1155	APA01_40500,<br>APA01_43100	NI	NI	b0698	KdpA

kdpB

Sme	Bja	Ccr	Pzu	Rsp	Rde	Sphm	Zmo	Rru	Apt	Rri	Wol	Eco	Pfam motif
SMa2331	bll6778	CC_1592	NI	RSP_1266	NI	G432_12250	NI	Rru_A1156	APA01_40510,<br>APA01_43110	NI	NI	b0697	E1-E2_ATPase Hydrolase

kdpC

Sme	Bja	Ccr	Pzu	Rsp	Rde	Sphm	Zmo	Rru	Apt	Rri	Wol	Eco	Pfam motif
SMa2329	bll6777	CC_1593	NI	RSP_1267	NI	G432_12255	NI	Rru_A1157	APA01_40520,<br>APA01_43120	NI	NI	b0696	KdpC

kdpD

Sme	Bja	Ccr	Pzu	Rsp	Rde	Sphm	Zmo	Rru	Apt	Rri	Wol	Eco	Pfam motif
SMa2327	bll6776	CC_1594	PHZ_c2647	RSP_1268	NI	G432_12260	NI	Rru_A1158	APA01_40530,<br>APA01_43130	NI	NI	b0695	KdpD, USP, DUF4118,<br>GFA, HisKA, HATPse_c

kdpE

Sme	Bja	Ccr	Pzu	Rsp	Rde	Sphm	Zmo	Rru	Apt	Rri	Wol	Eco	Pfam motif
SMa2325	bll6775	CC_1595	PHZ_c2648	RSP_1269	NI	G432_12265	NI	Rru_A1159	APA01_40540,<br>APA01_43140	NI	NI	b0694	Response_reg, Trans_reg_C

kdpF

Sme	Bja	Ccr	Pzu	Rsp	Rde	Sphm	Zmo	Rru	Apt	Rri	Wol	Eco	Pfam motif
NI	NI	NI	NI	NI	NI	NI	NI	NI	NI	NI	NI	b4513	Potass_KdpF

Glutamate synthesis system

glnA

Sme	Bja	Ccr	Pzu	Rsp	Rde	Sphm	Zmo	Rru	Apt	Rri	Wol	Eco	Pfam motif
SMc00948	blr4949	CC_1969	PHZ_c1572	RSP_0147	RD1_2569	G432_02715	ZMO0493	Rru_A2086	APA01_06310	NI	NI	b3870	Gln-synt_N, Gln-synt_C

gltB

Sme	Bja	Ccr	Pzu	Rsp	Rde	Sphm	Zmo	Rru	Apt	Rri	Wol	Eco	Pfam motif
SMc04028	blr7743	CC_3607	PHZ_c0164	RSP_1146	RD1_0060	G432_13005	ZMO1117	Rru_A0019	APA01_19810	NI	NI	b3212	GATase_2, Glu_syn_central,<br>Glu_synthase, GXGXG

gltD

Sme	Bja	Ccr	Pzu	Rsp	Rde	Sphm	Zmo	Rru	Apt	Rri	Wol	Eco	Pfam motif
SMc04026	blr7744	CC_3606	PHZ_c0165	RSP_1149	RD1_0063	G432_13000	ZMO1116	Rru_A0018	APA01_19820	NI	NI	b2468, b2887, b3213	Fer4_20, Pyr_redox_2

gdhA

Sme	Bja	Ccr	Pzu	Rsp	Rde	Sphm	Zmo	Rru	Apt	Rri	Wol	Eco	Pfam motif
Sma0228	NI	CC_2082	PHZ_c2888	RSP_0398	RD1_2450	G432_00795	NI	Rru_A1040	NI	NI	NI	b1761	ELFV_dehydrog_N, ELFV_dehydrog

Glycine betaine synthesis system

	Sme	Bja	Ccr	Pzu	Rsp	Rde	Sphm	Zmo	Rru	Apt	Rri	Wol	Eco	Pfam motif
betA	SMc00093	NI	CC_2642	PHZ_c1110	RSP_2184	RD1_2024	G432_02310	NI	NI	NI	NI	NI	b0311	GMC_oxred_N, GMC_oxred_C
betB	SMc00094	NI	NI	NI	RSP_2183	RD1_2023	NI	NI	NI	NI	NI	NI	b0312	Aldedh
betC	SMc00127	blr0426	NI	NI	RSP_0594	RD1_2022	NI	NI	NI	NI	NI	NI	b3678	Sulfatase Choline_sulf_C
betI	SMa1726	NI	NI	NI	RSP_2182	RD1_2021	NI	NI	NI	NI	NI	NI	b0313	TetR_N, TetR_C_6

Glycine betaine transport system

	Sme	Bja	Ccr	Pzu	Rsp	Rde	Sphm	Zmo	Rru	Apt	Rri	Wol	Eco	Pfam motif
betS	SM_b20333	NI	NI	PHZ_c1942	RSP_3080	RD1_2292	NI	NI	NI	NI	NI	NI	b0314	BCCT

Glycine betaine, proline and carnitine transport system

	Sme	Bja	Ccr	Pzu	Rsp	Rde	Sphm	Zmo	Rru	Apt	Rri	Wol	Eco	Pfam motif
proP	NI	NI	NI	PHZ_c1340	NI	NI	NI	NI	NI	APA01_21460	A1G_07490	WD0168	b4111	Sugar-tr, MSF_1, MSF_2
proV	SMc00670	NI	NI	NI	RSP_3057	RD1_2932	NI	NI	Rru_A2475	NI	NI	NI	b2677	ABC_trans
proW	SMc00671	NI	NI	NI	RSP_3999	RD1_2931	NI	NI	Rru_A2476	NI	NI	NI	b2678	BPD_transp_1
proX	SMc00672	NI	NI	NI	RSP_3998	RD1_1839	NI	NI	Rru_A2477	NI	NI	NI	b2679	OpuAC

Proline synthesis system

	Sme	Bja	Ccr	Pzu	Rsp	Rde	Sphm	Zmo	Rru	Apt	Rri	Wol	Eco	Pfam motif
proA	SMc03777	blr0429	CC_3430	PHZ-c0252	RSP_3824	RD1_2658	G432_04180	ZMO01661	Rru_A1238	APA01_03740	NI	NI	b0243	Aldedh
proB	SMc03776	blr0428	CC_0314	PHZ_c0253	RSP_3823	RD1_2659	G432_17290	ZMO0206	Rru_A1239	APA01_18840	NI	NI	b0242	AA_kinse, PUA
proC	SMc02677	blr7454	CC_0494	PHZ_c2855	RSP_3364	RD1_3497	G432_14415	ZMO0311	Rru_A3281	APA01_26410	NI	NI	b0386	F420_oxidored, P5CR-dimer

Trehalose synthesis system

	Sme	Bja	Ccr	Pzu	Rsp	Rde	Sphm	Zmo	Rru	Apt	Rri	Wol	Eco	Pfam motif
ostA	SMa0233	bll0322	NI	PHZ_c1816, PHZ_c3258	RSP_0948	NI	G432_06395	NI	Rru_A2485	APA01_02340	NI	NI	b1896	Glyco_transf_20
ostB	NI	bll0323	NI	PHZ_c3257	RSP_0949	NI	G432_06385	NI	Rru_A2549	APA01_02330	NI	NI	b1897	Trehalose_PPase

In silico analysis performed as described in the Methods. Sme, Bja, Ccr, Pzu, Rsp, Rde, Sphm, Zmo, Rru, Apt, Rri, Wol and Eco correspond to *Sinorhizobium meliloti* 1021, *Bradyrhizobium japonicum* USDA110, *Caulobacter crescentus* CB15, *Phenylobacterium zucineum*, *Rhodobacter sphaeroides* 2.4.1, *Roseobacter denitrificans*, *Sphingomonas sp*. MM-1, *Zymomonas mobilis* subsp. *mobilis* ZM4, *Rhodospirillum rubrum* ATCC11170, *Acetobacter pasteurianus* IFO 3283-01, *Rickettsia rickettsii* Sheila Smith, *Wolbachia sp*.wMel and *Escherichia coli*. K-12 MG1655, respectively. Accession numbers (locus number) are according to The Kyoto Encyclopedia of Genes and Genomes (KEGG). Protein domains were identified using The Pfam protein families database. NI refers to genes absent in the corresponding bacterium.
