# Supplementary material for: A comprehensive genomic, transcriptomic and proteomic analysis of a hyperosmotic stress sensitive α-proteobacterium
Source: BMC Microbiol. 2015 Mar 26;15:71. doi: 10.1186/s12866-015-0404-x (PMC4391529; doi:10.1186/s12866-015-0404-x)
Supplement: Additional file 2: Table S2. — Genes and proteins differentially expressed under hyperosmotic stress in Caulobacter crescentus. [file 12866_2015_404_MOESM2_ESM.pdf]

Table S2. Genes and proteins differentially expressed under hyperosmotic stress in *Caulobacter crescentus*

gene number[a]	transcriptomic[b] suc/ no stress	transcriptomic[b] NaCl/ no stress	proteomic[c] suc/ no stress	proteomic[c] NaCl/ no stress	carbon starvation[d]	regulator[e]	putative function[f]	functional category[g]	subcategory[g]	operon[h]
CC_0797	2.49	3.37	-	-			1,4-beta-D-glucan glucohydrolase D (celD) [3.2.1.74]	energy metabolism	sugar metabolism	
CC_0968	-	-	2.26	1.99			beta-D-glucosidase {3.2.1.21}	energy metabolism	sugar metabolism	
CC_1057	1.42	1.33	1.48	1.31			2-dehydro-3-deoxygluconokinase (kdgK) [2.7.1.45]	energy metabolism	Entner Doudoroff pathway	
CC_1135	5.54	-	-	-			amylosucrase [2.4.1.4]	energy metabolism	sugar metabolism	CC1133-CC1135
CC_1571	1.14	-	-	-			gluconate 5-dehydrogenase (gno) [1.1.1.69]	energy metabolism	gluconate metabolism	CC1567-CC1571
CC_1630	1.94	-	-	-			glucose-fructose oxidoreductase (EC:1.1.99.28)	energy metabolism	enzymes of unknown specificity	CC1628-CC1632
CC_1632	2.23	-	-	-			cytochrome c family protein	energy metabolism	electron transport	CC1628-CC1632
CC_1635	2.13	-	-	-			gluconate 2-dehydrogenase subunit 3	energy metabolism	gluconate metabolism	CC1634-CC1635
CC_1696	-	1.13	-	-			cytochrome c family protein	energy metabolism	electron transport	
CC_2055	1.38	-	1.43	1.37			phosphogluconate dehydratase (edd) [4.2.1.12]	energy metabolism	Entner Doudoroff pathway	CC2054-CC2057
CC_2057	-	-	1.02	1.14			glucose-6-phosphate 1-dehydrogenase (zwf) [1.1.1.49]	energy metabolism	Entner Doudoroff pathway	
CC_2282	-	-	2.40	-			glucoamylase (cga) [3.2.1.3]	energy metabolism	sugar metabolism	
CC_3054	-	-	1.14	1.28			xylosidase/arabinosidase (xarB)	energy metabolism	sugar metabolism	
CC_3167	1.27	-	-	-			glucokinase {2.7.1.2}	energy metabolism	glycolysis/gluconeogenesis	CC3167-CC3168
CC_0047	-	-2.02	-	-			inorganic pyrophosphatase (ppa) [3.6.1.1]	energy metabolism	oxidative phosphorylation	
CC_0365	-	-1.66	-	-			ATP synthase F0, B subunit (atpF) [3.6.3.14]	energy metabolism	oxidative phosphorylation	CC0365-CC0369
CC_0366	-	-1.70	-	-			ATP synthase F0, B subunit (atpF) [3.6.3.14]	energy metabolism	oxidative phosphorylation	CC0365-CC0369
CC_0368	-1.38	-2.20	-	-			ATP synthase F0, A subunit (atpB) [3.6.3.14]	energy metabolism	oxidative phosphorylation	CC0365-CC0369
CC_0472	-	-1.16	-	-			ubiquinol-cytochrome c reductase, iron-sulfur subunit (petA) [1.10.2.2]	energy metabolism	oxidative phosphorylation	CC0742-CC0744
CC_1471	-1.44	-	-1.41	-1.64			pyruvate phosphate dikinase (ppdK) [2.7.9.1]	energy metabolism	glycolysis/gluconeogenesis	
CC_1495	-	-1.37	-	-			keto-hydroxyglutarate-aldolase/keto-deoxy-phosphogluconate aldolase (4.1.2.14/ 4.1.3.16)	energy metabolism	Entner Doudoroff pathway	CC1495-CC1496
CC_1496	-	-1.36	-	-			carbohydrate kinase {2.7.1.45}	energy metabolism	Entner Doudoroff pathway	CC1495-CC1496
CC_1724	-	-1.84	-	-			enolase (eno) [4.2.1.11]	energy metabolism	glycolysis/gluconeogenesis	
CC_1726	-	-	-1.11	-1.06			pyruvate dehydrogenase complex, E1 component, pyruvate dehydrogenase alpha subunit (pdhA) [1.2.4.1]	energy metabolism	glycolysis/gluconeogenesis	
CC_1727	-	-1.77	-1.27	-1.37			pyruvate dehydrogenase complex, E1 component, pyruvate dehydrogenase beta subunit (pdhB) [1.2.4.1]	energy metabolism	glycolysis/gluconeogenesis	CC1726-CC1727
CC_1729	-	-1.60	-	-			pyruvate dehydrogenase complex, E2 component, dihydrolipoamide acetyltransferase (aceF) [2.3.1.12]	energy metabolism	glycolysis/gluconeogenesis	CC1729-CC1730
CC_1731	-	-1.64	-	-			pyruvate dehydrogenase complex, E3 component, lipoamide dehydrogenase (lpdA) [1.8.1.4]	energy metabolism	glycolysis/gluconeogenesis	
CC_1770	-2.64	-2.84	-	-			ubiquinol oxidase subunit IV (qoxD)	energy metabolism	oxidative phosphorylation	CC1767-CC1773
CC_1771	-3.48	-3.94	-	-			ubiquinol oxidase subunit III (qoxC)	energy metabolism	oxidative phosphorylation	CC1767-CC1773
CC_1772	-3.63	-4.40	-	-			ubiquinol oxidase subunit I (qoxB)	energy metabolism	oxidative phosphorylation	CC1767-CC1773
CC_1906	-	-1.78	-	-			citrate synthase (gltA) [2.3.3.1]	energy metabolism	tricarboxylic acid cycle	
CC_1938	-	-1.39	-	-			NADH dehydrogenase I, M subunit (nuoM) [1.6.5.3]	energy metabolism	oxidative phosphorylation	CC1933--CC1952
CC_1939	-	-1.30	-	-			NADH dehydrogenase I, L subunit (nuoL) [1.6.5.3]	energy metabolism	oxidative phosphorylation	CC1933--CC1952
CC_1940	-	-1.27	-	-			NADH dehydrogenase I, K subunit (nuoK) [1.6.5.3]	energy metabolism	oxidative phosphorylation	CC1933--CC1952
CC_1942	-	-1.25	-	-			NADH dehydrogenase I, I subunit (nuoI) [1.6.5.3]	energy metabolism	oxidative phosphorylation	CC1933--CC1952
CC_1950	-	-1.26	-	-			NADH dehydrogenase I, E subunit (nuoE) [1.6.5.3]	energy metabolism	oxidative phosphorylation	CC1933--CC1952
CC_2261	-	-1.63	-	-			phosphoglycerate mutase (gpm) [5.4.2.1]	energy metabolism	glycolysis/gluconeogenesis	
CC_3248	-	-1.76	-1.07	-			glyceraldehyde 3-phosphate dehydrogenase (gap) [1.2.1.12]	energy metabolism	glycolysis/gluconeogenesis	CC3248-CC3250
CC_3250	-2.74	-3.39	-	-			fructose-bisphosphate aldolase, class II (fbaA) [4.1.2.13]	energy metabolism	glycolysis/gluconeogenesis	CC3248-CC3250
CC_3445	-	-1.68	-	-			ATP synthase F1, epsilon subunit (atpC) [3.6.3.14]	energy metabolism	oxidative phosphorylation	CC3445-CC3450
CC_3447	-	-	-1.75	-1.41			ATP synthase F1, beta subunit (atpD) [3.6.3.14]	energy metabolism	oxidative phosphorylation	CC3445-CC3450
CC_3448	-	-1.79	-	-			ATP synthase F1, gamma subunit (atpG) [3.6.3.14]	energy metabolism	oxidative phosphorylation	CC3445-CC3450
CC_3449	-	-1.52	-1.04	-			ATP synthase F1, alpha subunit (atpA) [3.6.3.14]	energy metabolism	oxidative phosphorylation	CC3445-CC3450
CC_1344	1.42	1.96	-	-			predicted metal-dependent hydrolase	central intermediary metabolism	enzymes of unknown specificity	
CC_1634	4.60	3.23	1.67	-			glucose-methanol-choline oxidoreductase	central intermediary metabolism	enzymes of unknown specificity	CC1634-CC1635
CC_3428	-	-	2.70	-			oxidoreductase, aldo/keto reductase family	central intermediary metabolism	enzymes of unknown specificity	
CC_3533	-	1.31	-	-			acyltransferase family protein	central intermediary metabolism	enzymes of unknown specificity	CC3533-CC3538
CC_3759	2.54	1.55	-	-		SigT	quinone oxidoreductase (qor) [1.6.5.5]	central intermediary metabolism	enzymes of unknown specificity	CC3758-CC3759
CC_0213	-	-1.42	-	-			acetyltransferase, GNAT family	central intermediary metabolism	enzymes of unknown specificity	CC0211-CC0213
CC_0282	-	-1.83	-	-			hydrolase, haloacid dehalogenase-like family	central intermediary metabolism	enzymes of unknown specificity	CC0281-CC0283
CC_0654	-1.72	-2.26	-	-			ferredoxin A (fdxA)	central intermediary metabolism	electron transport	
CC_1119	-4.91	-5.43	-	-			sulfite reductase (NADPH) hemoprotein beta-component (cysI) [1.8.1.2]	central intermediary metabolism	sulfur metabolism	CC1119-CC1121
CC_1121	-3.25	-	-	-			phospho-adenylylsulfate reductase (cysH) [1.8.4.8]	central intermediary metabolism	sulfur metabolism	CC1119-CC1121
CC_1295	-1.72	-1.72	-	-			3-phytase, fusion, putative {3.1.3.8}	central intermediary metabolism	phosphate availability	
CC_1482	-3.78	-3.66	-	-			sulfate adenylate transferase, subunit 1/adenylylsulfate kinase (cysN/C) [2.7.1.25/2.7.7.4]	central intermediary metabolism	sulfur metabolism	CC1482-CC1483
CC_1483	-3.52	-3.36	-	-			sulfate adenylate transferase, subunit 2 (cysD) [2.7.7.4]	central intermediary metabolism	sulfur metabolism	CC1482-CC1483
CC_1864	-	-1.84	-	-			cysteine desulfurase (sufB)	central intermediary metabolism	Fe-S cluster assembly protein	CC1863-CC1866
CC_2096	-	-1.16	-	-			hydrolase, haloacid dehalogenase-like family	central intermediary metabolism	enzymes of unknown specificity	CC2096-CC2097

CC_3208	-1.63	-2.17	-	-	ferredoxin-NADP reductase {1.18.1.2}	central intermediary metabolism	electron transport	
CC_3320	-	-1.50	-	-	hydrolase, haloacid dehalogenase-like family	central intermediary metabolism	enzymes of unknown specificity	CC3320-CC3321
CC_3539	-1.54	-	-	-	thioredoxin	central intermediary metabolism	electron transport	
CC_2577	-	2.57	-	-	pyridoxamine 5'-phosphate oxidase	cofactor metabolism	vitamin B6 metabolism	
CC_3303	1.13	-	-	-	NAD(P) transhydrogenase, beta subunit (pntB) [1.6.1.2]	cofactor metabolism	nicotinate and nicotinamide metabolism	CC3303-CC3305
CC_0887	-2.15	-1.64	-	-	3,4-dihydroxy-2-butanone 4-phosphate synthase/GTP cyclohydrolase II (ribAB) [4.1.99.12/ 3.5.4.25]	cofactor metabolism	riboflavin, FAD and FMN metabolism	CC0887-CC0888
CC_1118	-3.19	-3.13	-	-	uroporphyrin-III C-methyltransferase [2.1.1.107]	cofactor metabolism	porphyrin metabolism	
CC_1577	-	-1.31	-	-	adenosylmethionine-8-amino-7-oxononanoate aminotransferase (bioA) [2.6.1.62]	cofactor metabolism	biotin metabolism	
CC_1935	-	-1.22	-	-	pantothenate kinase {2.7.1.33}	cofactor metabolism	coenzyme A synthesis	CC1933--CC1952
CC_2140	-3.33	-	-	-	5,10-methylenetetrahydrofolate reductase (metF) [1.5.1.20]	cofactor metabolism	folate metabolism	CC2137-CC2141
CC_3355	-1.96	-2.67	-	-	glycine cleavage system T protein (gcvT) [2.1.2.10]	cofactor metabolism	folate metabolism	CC3353-CC3355
CC_3630	-	-1.42	-	-	formyltetrahydrofolate deformylase (purU) [3.5.1.10]	cofactor metabolism	folate metabolism	
CC_2274	-	-	1.40	1.54	malonate-semialdehyde dehydrogenase/methylmalonate-semialdehyde dehydrogenase {1.2.1.18/ 1.2.1.27}	amino acid metabolism	valine and isoleucine degradation	
CC_3143	-	-	2.64	2.52	omega-amino acid-pyruvate aminotransferase [2.6.1.18]	amino acid metabolism	valine and isoleucine degradation	
CC_0050	-3.22	-4.75	-	-	S-adenosylmethionine synthetase (metK) [2.5.1.6]	amino acid metabolism	cysteine and methionine metabolism	CC0049-CC0050
CC_0193	-	-1.25	-	-	3-isopropylmalate dehydrogenase (leuB) [1.1.1.85]	amino acid metabolism	leucine, isoleucine and valine metabolism	CC0193-CC0194
CC_0211	-	-1.45	-	-	agmatine deiminase {3.5.3.12}	amino acid metabolism	arginine and proline metabolism	CC0211-CC0213
CC_0212	-1.12	-1.89	-	-	N-carbamoylputrescine amidase {3.5.1.53}	amino acid metabolism	arginine and proline metabolism	CC0211-CC0213
CC_0253	-1.44	-2.02	-	-	aspartate-semialdehyde dehydrogenase (asd) [1.2.1.11]	amino acid metabolism	aspartate metabolism	
CC_0257	-3.22	-4.64	-2.50	-2.42	adenosylhomocysteinase (achY) [3.3.1.1]	amino acid metabolism	cysteine and methionine metabolism	CC00255-CC0257
CC_0360	-1.91	-2.61	-	-	ornithine decarboxylase {4.1.1.17}	amino acid metabolism	arginine and proline metabolism	
CC_0482	-2.90	-2.95	-2.40	-2.47	5-methyltetrahydropteroyltriglutamate-homocysteine methyltransferase (metE) [2.1.1.14]	amino acid metabolism	cysteine and methionine metabolism	
CC_1969	-1.89	-3.28	-2.02	-2.05	glutamine synthetase, type I (glnA) [6.3.1.2]	amino acid metabolism	glutamate metabolism	CC1968-CC1969
CC_2082	-	-1.18	-	-	Glu/Leu/Phe/Val dehydrogenase {1.4.1.9}	amino acid metabolism	glutamate metabolism	
CC_2137	-3.34	-3.94	-	-	5-methyltetrahydrofolate-homocysteine methyltransferase (metH) [2.1.1.13]	amino acid metabolism	cysteine and methionine metabolism	CC2137-CC2141
CC_2138	-	-4.05	-	-	5-methyltetrahydrofolate-homocysteine methyltransferase (metH) [2.1.1.13]	amino acid metabolism	cysteine and methionine metabolism	CC2137-CC2141
CC_2211	-	-1.50	-	-	argininosuccinate lyase (argH) [4.3.2.1]	amino acid metabolism	arginine and proline metabolism	
CC_2223	-	-	-1.61	-1.39	histidinol-phosphate aminotransferase (hisC) [2.6.1.9]	amino acid metabolism	histidine metabolism	
CC_2238	-	-1.48	-	-	O-succinylhomoserine sulfhydrylase (metZ) [2.5.1.-]	amino acid metabolism	cysteine and methionine metabolism	CC2238-CC2239
CC_3044	-	-	-	-1.15	dihydroxy-acid dehydratase (ilvD) [4.2.1.9]	amino acid metabolism	leucine, isoleucine and valine metabolism	
CC_3215	-2.00	-	-	-	D-3-phosphoglycerate dehydrogenase (serA) [1.1.1.95]	amino acid metabolism	serine metabolism	CC3215-CC3216
CC_3216	-2.02	-2.54	-1.36	-1.73	phosphoserine aminotransferase (serC) [2.6.1.52]	amino acid metabolism	serine metabolism	CC3215-CC3216
CC_3606	-2.22	-2.69	-2.06	-1.95	glutamate synthase, small subunit (gltD) [1.4.1.13]	amino acid metabolism	glutamate metabolism	CC3606-CC3607
CC_3607	-	-	-2.64	-2.22	glutamate synthase, large subunit (gltB) [1.4.1.13]	amino acid metabolism	glutamate metabolism	
CC_3734	-1.63	-2.33	-	-	imidazoleglycerol-phosphate dehydratase (hisB) [4.2.1.19]	amino acid metabolism	histidine metabolism	CC3733-CC3738
CC_2796	-	1.91	-	-	aminoacrylate peracid reductase	nucleotide metabolism	pyrimidine metabolism	CC2795-CC2996
CC_2797	-	1.41	-	-	aminoacrylate hydrolase {3.5.1.-}	nucleotide metabolism	pyrimidine metabolism	
CC_2798	1.09	1.87	-	-	pyrimidine oxygenase {1.14.99.46}	nucleotide metabolism	pyrimidine metabolism	
CC_0086	-2.30	-	-	-	phosphoribosylaminoimidazolecarboxamide formyltransferase/IMP cyclohydrolase (purH) [2.1.2.3/ 3.5.4.10]	nucleotide metabolism	purine and folate biosynthesis	CC0086-CC0087
CC_1620	-	-1.41	-	-	GMP synthase (guaA) [6.3.5.2]	nucleotide metabolism	purine biosynthesis	
CC_1699	-1.32	-1.93	-	-	nucleoside diphosphate kinase (ndk) [2.7.4.6]	nucleotide metabolism	purine and pyrimidine biosynthesis	
CC_2491	-	-1.07	-	-	phosphoribosylaminoimidazole-succinocarboxamide synthase (purC) [6.3.2.6]	nucleotide metabolism	purine biosynthesis	CC2491-CC2492
CC_2900	-	-2.05	-1.80	-2.19	carbamoyl-phosphate synthase, large subunit (carB) [6.3.5.5]	nucleotide metabolism	pyrimidine metabolism	
CC_3103	-	-1.10	-	-	adenylosuccinate synthetase (purA) [6.3.4.4]	nucleotide metabolism	purine biosynthesis	
CC_0076	-	-	1.25	1.01	3-hydroxyacyl-CoA dehydrogenase /enoyl-CoA hydratase/ 3-hydroxybutyryl-CoA epimerase (fadB) {1.1.1.35/ 4.2.1.17/ 5.1.2.3}	fatty acid and polyhydroxyalkanoate metabolism	fatty acid and polyhydroxyalkanoate metabolism	
CC_0353	-	1.60	-	-	enoyl-CoA hydratase {4.2.1.17}	fatty acid and polyhydroxyalkanoate metabolism	fatty acid and polyhydroxyalkanoate metabolism	
CC_0427	1.24	1.66	-	-	acyl-CoA dehydrogenase (mmgC) [1.3.99.-]	fatty acid and polyhydroxyalkanoate metabolism	fatty acid metabolism	
CC_0510	1.21	1.83	1.58	1.68	acetyl-CoA acetyltransferase (phbA) [2.3.1.9]	fatty acid and polyhydroxyalkanoate metabolism	polyhydroxyalkanoate metabolism	CC0510-CC0511
CC_0511	-	1.93	2.31	2.44	acetoacetyl-CoA reductase (phbB) [1.1.1.36]	fatty acid and polyhydroxyalkanoate metabolism	polyhydroxyalkanoate metabolism	CC0510-CC0511
CC_0922	1.26	-	-	-	fatty-acyl-CoA synthase [6.2.1.-]	fatty acid and polyhydroxyalkanoate metabolism	fatty acid metabolism	
CC_0942	-	1.76	2.93	2.76	acyl dehydratase MaoC	fatty acid and polyhydroxyalkanoate metabolism	fatty acid metabolism	
CC_0944	3.07	3.20	-	-	acyl-CoA synthetase {6.2.1.3}	fatty acid and polyhydroxyalkanoate metabolism	fatty acid metabolism	
CC_0947	1.41	1.94	-	-	enoyl-CoA hydratase {4.2.1.17}	fatty acid and polyhydroxyalkanoate metabolism	fatty acid metabolism	
CC_1311	-	1.14	1.33	-	acyl-CoA dehydrogenase family protein {1.3.99.-}	fatty acid and polyhydroxyalkanoate metabolism	fatty acid metabolism	CC1309-CC1311
CC_1380	-	1.58	-	-	poly-beta-hydroxybutyrate polymerase (phbC) [2.3.1.-]	fatty acid and polyhydroxyalkanoate metabolism	polyhydroxyalkanoate metabolism	
CC_2254	-	1.14	-	-	SCP-2 sterol transfer family	fatty acid and polyhydroxyalkanoate metabolism	fatty acid metabolism	
CC_2405	1.30	1.77	-	-	3-oxoadipate CoA-transferase, alpha subunit (pcaI) [2.8.3.6]	fatty acid and polyhydroxyalkanoate metabolism	polyhydroxyalkanoate metabolism	CC2404-CC2411
CC_3454	1.95	-	-	-	acyl-CoA dehydrogenase [1.3.8.7]	fatty acid and polyhydroxyalkanoate metabolism	fatty acid, amino acid metabolism	

CC_0814	1.94	2.08	-	-		major facilitator family transporter	transport	transporter protein of unknown specificity	
CC_0970	-	3.19	-	-		TonB-dependent receptor	transport	transporter protein of unknown specificity	
CC_0991	2.23	2.82	1.76	2.38		TonB-dependent receptor	transport	transporter protein of unknown specificity	
CC_0999	1.63	2.26	-	-		TonB-dependent receptor, putative	transport	transporter protein of unknown specificity	
CC_1099	-	1.47	-	-		TonB-dependent receptor	transport	transporter protein of unknown specificity	
CC_1103	1.78	1.97	-	-		sugar MFS transporter, fucose:H+ symporter (FHS) family	transport	transporter for sugar	
CC_1133	4.81	-	-	-		major facilitator family transporter	transport	transporter protein of unknown specificity	CC1133-CC1135
CC_1136	7.01	-	5.50	-		TonB-dependent receptor	transport	transporter protein of unknown specificity	
CC_1275	-	1.80	-	-		ABC-2 type transport system permease protein	transport	transporter protein of unknown specificity	CC1275-CC1276
CC_1363	2.77	3.25	-	-		V-type H(+)-translocating pyrophosphatase [3.6.1.1]	transport	transporter for proton	
CC_1628	2.54	-	-	-		major facilitator family transporter	transport	transporter protein of unknown specificity	CC1628-CC1632
CC_1750	-	-	-	1.03		TonB-dependent receptor	transport	transporter protein of unknown specificity	
CC_1774	2.38	2.66	-	-		major facilitator family transporter	transport	transporter protein of unknown specificity	
CC_2091	-	1.69	-	-		ABC transport system ATP-binding protein	transport	transporter protein of unknown specificity	CC2090-CC2092
CC_2287	2.35	-	2.61	3.60		TonB-dependent receptor	transport	transporter protein of unknown specificity	
CC_2320	1.72	1.47	-	-		ABC transporter, periplasmic substrate-binding protein, putative	transport	transporter protein of unknown specificity	CC2318-CC2321
CC_2347	-	1.98	-	-		cytosine/purines/uracil/thiamine/allantoin permease family protein	transport	transporter for purines and pyrimidines	
CC_2485	2.35	2.95	-	-		major facilitator family transporter	transport	transporter protein of unknown specificity	CC2485-CC2486
CC_3127	-	2.14	1.12	2.05		TonB-dependent receptor	transport	transporter protein of unknown specificity	CC3125-CC2128
CC_3161	3.52	4.61	-	-		TonB-dependent receptor	transport	transporter protein of unknown specificity	
CC_3336	1.73	2.78	-	-		TonB-dependent receptor, putative	transport	transporter protein of unknown specificity	CC3336-CC3337
CC_3461	3.08	3.81	-	-		TonB-dependent receptor	transport	transporter protein of unknown specificity	
CC_0254	-1.36	-2.09	-	-		transporter, chloramphenicol-sensitivity protein (RarD) family (rarD)	transport	detoxification	
CC_0286	-2.66	-2.70	-	-		sulfate transport system substrate-binding protein	transport	transporter for sulfate	
CC_1517	-3.75	-3.19	-	-		TonB-dependent receptor	transport	transporter protein of unknown specificity	
CC_1518	-3.58	-3.61	-	-		ABC transporter, ATP-binding protein	transport	transporter protein of unknown specificity	
CC_1519	-3.43	-3.35	-	-		ABC-2 type transport system permease protein	transport	transporter protein of unknown specificity	
CC_1597	-1.21	-	-	-		sulfate ABC transporter, permease protein (cysW)	transport	transporter for sulfate	CC1596-CC1602
CC_1598	-2.84	-3.07	-	-		sulfate ABC transporter, ATP-binding protein (cysA) [3.6.3.25]	transport	transporter for sulfate	CC1596-CC1602
CC_1666	-1.58	-1.20	-	-		TonB-dependent receptor	transport	transporter protein of unknown specificity	
CC_1778	-5.32	-4.58	-	-		TonB-dependent receptor	transport	transporter protein of unknown specificity	CC1777-CC1778
CC_1781	-3.24	-3.54	-	-		TonB-dependent receptor	transport	transporter protein of unknown specificity	CC1781-CC1782
CC_1862	-	-1.11	-	-		ABC transporter, ATP-binding protein	transport	transporter protein of unknown specificity	CC1857-CC1862
CC_1970	-	-	-1.67	-		TonB-dependent receptor	transport	transporter protein of unknown specificity	
CC_2148	-	-1.61	-	-		ABC transporter, ATP-binding protein	transport	transporter protein of unknown specificity	
CC_2664	-1.57	-	-	-		methionine ABC transporter, periplasmic methionine-binding protein (metQ)	transport	transporter for methionine	
CC_2669	-	-1.09	-	-		methionine ABC transporter, ATP-binding protein (metN)	transport	transporter for methionine	CC2668-CC2669
CC_2928	-	-1.58	-	-		TonB-dependent receptor	transport	transporter protein of unknown specificity	CC2927-CC2928
CC_3013	-1.38	-2.44	-	-		TonB-dependent receptor	transport	transporter protein of unknown specificity	

CC_0063	-	1.58	-	-		cytochrome P450 family protein	environmental adaptation	detoxification	
CC_0419	-	-	1.18	1.20		aldehyde dehydrogenase {1.2.1.3}	environmental adaptation	detoxification	
CC_0557	-	1.38	-	-	SigT	ferritin-like protein	environmental adaptation	iron storage	CC0555-CC0557
CC_0941	-	1.45	-	-		OsmC-like protein	environmental adaptation	unknown function	
CC_1178	3.76	3.72	-	-	SigT/SigU	stress-induced bacterial acidophilic repeat motif	environmental adaptation	unknown function	CC1178-CC1179
CC_1452	2.82	3.24	-	-	SigT	universal stress protein family	environmental adaptation	unknown function	CC1452-CC1453
CC_1533	1.34	-	-	-	SigT	glutathione S-transferase {2.5.1.18}	environmental adaptation	detoxification	
CC_1849	2.14	-	-	-		coniferyl aldehyde dehydrogenase {1.2.1.68}	environmental adaptation	xenobiotics biodegradation	
CC_2281	1.06	-	-	-		methyl-accepting chemotaxis protein McpE (mcpE)	environmental adaptation	chemotaxis and motility	CC2281-CC2283
CC_2409	-	1.55	-	-		protocatechuate 3,4-dioxygenase, alpha subunit (pcaG) [1.13.11.3]	environmental adaptation	xenobiotics biodegradation	CC2404-CC2411
CC_2515	2.40	1.67	-	-		S-formylglutathione hydrolase {3.1.2.12}	environmental adaptation	xenobiotics biodegradation	
CC_2516	1.42	-	2.49	1.99		S-(hydroxymethyl)glutathione dehydrogenase /alcohol dehydrogenase [1.1.1.284/ 1.1.1.1]	environmental adaptation	detoxification	
CC_2644	3.83	5.49	-	-		PhoH family protein	environmental adaptation	adaptations to atypical conditions	
CC_2843	1.30	-	-	-		glutathione S-transferase {2.5.1.18}	environmental adaptation	detoxification	
CC_2873	2.07	-	-	-	SigT/SigU	starvation-inducible DNA-binding protein	environmental adaptation	DNA protection	CC2872-CC2873
CC_2997	-	1.51	-	-		cytochrome P450 family protein	environmental adaptation	detoxification	CC2997-CC2998
CC_3029	-	1.79	-	-		S-(hydroxymethyl)glutathione dehydrogenase /alcohol dehydrogenase [1.1.1.284/ 1.1.1.1]	environmental adaptation	detoxification	CC3029-CC3030
CC_3043	2.59	2.89	-	-		catalase-peroxidase (katG) [1.11.1.21]	environmental adaptation	detoxification	
CC_3088	1.23	1.76	-	-		glutathione S-transferase {2.5.1.18}	environmental adaptation	detoxification	
CC_3262	-	1.27	-	-		bacterioferritin (bfr)	environmental adaptation	iron storage	CC3262-CC3263
CC_3580	3.21	3.50	3.23	2.91		aldehyde dehydrogenase [1.2.1.3]	environmental adaptation	detoxification	
CC_3758	2.55	1.88	-	-	SigT	carboxymethylenebutenolidase (ysgA) [3.1.1.45]	environmental adaptation	xenobiotics biodegradation	CC3758-CC3759

CC_0575	-	-1.25	-	-		beta-lactamase, putative	environmental adaptation	detoxification	
CC_1730	-	-1.60	-	-		glutathione peroxidase [1.11.1.9]	environmental adaptation	detoxification	CC1729-CC1730
CC_1777	-4.28	-4.07	-	-		superoxide dismutase, Mn (sodA) [1.15.1.1]	environmental adaptation	detoxification	CC1777-CC1778
CC_1934	-	-1.39	-	-		metallo-beta-lactamase family protein	environmental adaptation	detoxification	CC1933--CC1952
CC_2139	-2.82	-3.30	-	-		metallo-beta-lactamase superfamily protein	environmental adaptation	detoxification	CC2137-CC2141
CC_2918	-1.59	-2.39	-1.63	-1.56		alkyl hydroperoxide reductase, subunit c (ahpC) [1.11.1.15]	environmental adaptation	detoxification	CC2918-CC2919
CC_2919	-	-1.30	-	-		alkyl hydroperoxide reductase, subunit f (ahpF) [1.11.1.15]	environmental adaptation	detoxification	CC2918-CC2919

CC_0201	1.76	1.59	-	-	SigT/SigU	OmpA family protein	cell envelope	surface structure	CC0201-CC0203
CC_0747	1.97	-	-	-	SigT/SigU	OmpA family protein	cell envelope	surface structure	
CC_1007	1.67	1.45	-	-		S-layer protein RsaA (rsaA)	cell envelope	surface structure	
CC_1695	-	1.54	-	-		CsgG containing protein	cell envelope	unknown function	
CC_1958	1.65	1.57	-	-	SigT	glycosyl transferase, group 1 family protein	cell envelope	biosynthesis and degradation of surface sugars	
CC_2294	-	-	1.63	-		outer membrane protein (omp28)	cell envelope	unknown function	
CC_3229	1.72	1.89	-	-		OmpA family protein	cell envelope	surface structure	CC3226-CC3229
CC_3489	1.76	1.90	-	-		penicillin-binding protein AmpH, putative	cell envelope	biosynthesis and degradation of surface sugars	
CC_1782	-3.31	-3.34	-	-		PepSY-associated TM helix domain protein	cell envelope	unknown function	CC1781-CC1782
CC_3501	-4.43	-4.59	-	-		PepSY-associated TM helix domain protein	cell envelope	unknown function	
CC_3629	-	-1.25	-	-		dTDP-D-glucose-4,6-dehydratase (rfbB) [4.2.1.46]	cell envelope	biosynthesis and degradation of surface sugars	

CC_0624	1.43	1.68	-	-		ISCc3, transposase OrfB	motile and extrachromosomal element functions	transposon functions	CC0624-CC0625
CC_2200	2.31	3.19	-	-		HNH endonuclease family protein	motile and extrachromosomal element functions	transposon functions	
CC_2681	-	1.42	-	-		integrase/recombinase, phage integrase family	motile and extrachromosomal element functions	prophage functions	

CC_3597	2.59	3.34	3.23	3.37		ribosomal subunit interface protein (yfiA)	protein synthesis	hibernation factor	CC3596-CC3597
CC_0035	-	-2.00	-	-		ribosomal protein S15 (rpsO)	protein synthesis	ribosomal protein	CC0035-CC0037
CC_0036	-	-1.39	-	-		tRNA pseudouridine synthase B (truB) [4.2.1.70]	protein synthesis	tRNAmodification	CC0035-CC0037
CC_0037	-1.29	-1.92	-	-		ribosome-binding factor A (rbfA)	protein synthesis	ribosome biogenesis	CC0035-CC0037
CC_0049	-2.55	-3.67	-	-		tRNA (guanine-N(7))-methyltransferase {2.1.1.33}	protein synthesis	tRNAmodification	CC0049-CC0050
CC_0197	-	-1.36	-	-		ribosomal protein L19 (rplS)	protein synthesis	ribosomal protein	CC0197-CC0198
CC_0318	-	-1.57	-	-		ribosomal protein L27 (rpmA)	protein synthesis	ribosomal protein	CC0318-CC0319
CC_0319	-	-1.68	-	-		ribosomal protein L21 (rplU)	protein synthesis	ribosomal protein	CC0318-CC0319
CC_0460	-	-1.51	-	-		cysteinyl-tRNA synthetase (cysS) [6.1.1.16]	protein synthesis	tRNA aminoacylation	CC0460-CC0462
CC_0485	-	-	-1.85	-2.92		ribosomal 5S rRNA E-loop binding protein Ctc/L25/TL5	protein synthesis	ribosomal protein	
CC_0496	-2.08	-4.02	-8.50	-1.47		ribosomal protein L10 (rplJ)	protein synthesis	ribosomal protein	CC0496-CC0497
CC_0497	-1.67	-	-	-		ribosomal protein L7/L12 (rplL)	protein synthesis	ribosomal protein	CC0496-CC0497
CC_0640	-1.47	-2.17	-1.67	-1.01		ribosomal protein L1 (rplA)	protein synthesis	ribosomal protein	CC0640-CC0641
CC_0641	-	-2.68	-	-		ribosomal protein L11 (rplK)	protein synthesis	ribosomal protein	CC0640-CC0641
CC_0661	-	-1.14	-	-		ribosomal protein L28 (rpmB)	protein synthesis	ribosomal protein	
CC_0701	-	-1.43	-1.49	-1.31		isoleucyl-tRNA synthetase (ileS) [6.1.1.5]	protein synthesis	tRNA aminoacylation	
CC_0721	-	-1.79	-	-		translation elongation factor P (efp)	protein synthesis	translation factors	
CC_0741	-1.29	-2.48	-	-1.09		elongation factor	protein synthesis	translation factors	
CC_1045	-	-1.81	-	-		ribosomal protein L20 (rplT)	protein synthesis	ribosomal protein	CC1045-CC1046
CC_1046	-	-1.70	-	-		ribosomal protein L35 (rpmI)	protein synthesis	ribosomal protein	CC1045-CC1046
CC_1196	-	-1.32	-	-		small protein B (smpB)	protein synthesis	protein quality control	CC1195-CC1196
CC_1240	-	-1.91	-	-		translation elongation factor EF-Tu (tufB)	protein synthesis	translation factors	
CC_1247	-1.53	-2.76	-	-		ribosomal protein S10 (rpsJ)	protein synthesis	ribosomal protein	CC1247-CC1267
CC_1248	-	-2.81	-2.12	-1.60		ribosomal protein L3 (rplC)	protein synthesis	ribosomal protein	CC1247-CC1267
CC_1249	-1.40	-2.97	-	-		ribosomal protein L4 (rplD)	protein synthesis	ribosomal protein	CC1247-CC1267
CC_1250	-1.32	-2.74	-	-		ribosomal protein L23 (rplW)	protein synthesis	ribosomal protein	CC1247-CC1267
CC_1251	-1.55	-2.79	-	-		ribosomal protein L2 (rplB)	protein synthesis	ribosomal protein	CC1247-CC1267
CC_1252	-1.68	-3.46	-	-		ribosomal protein S19 (rpsS)	protein synthesis	ribosomal protein	CC1247-CC1267
CC_1253	-1.91	-3.03	-	-		ribosomal protein L22 (rplV)	protein synthesis	ribosomal protein	CC1247-CC1267
CC_1254	-1.60	-3.22	-	-		ribosomal protein S3 (rpsC)	protein synthesis	ribosomal protein	CC1247-CC1267
CC_1255	-1.72	-3.16	-	-		ribosomal protein L16 (rplP)	protein synthesis	ribosomal protein	CC1247-CC1267
CC_1256	-1.80	-3.53	-	-		ribosomal protein L29 (rpmC)	protein synthesis	ribosomal protein	CC1247-CC1267
CC_1257	-1.95	-3.68	-	-		ribosomal protein S17 (rpsQ)	protein synthesis	ribosomal protein	CC1247-CC1267
CC_1258	-1.85	-3.59	-	-		ribosomal protein L14 (rplN)	protein synthesis	ribosomal protein	CC1247-CC1267
CC_1259	-1.94	-3.71	-	-		ribosomal protein L24 (rplX)	protein synthesis	ribosomal protein	CC1247-CC1267
CC_1260	-1.94	-3.88	-2.30	-1.36		ribosomal protein L5 (rplE)	protein synthesis	ribosomal protein	CC1247-CC1267
CC_1261	-1.69	-3.34	-	-		ribosomal protein S14 (rpsN)	protein synthesis	ribosomal protein	CC1247-CC1267
CC_1262	-1.83	-3.30	-	-		ribosomal protein S8 (rpsH)	protein synthesis	ribosomal protein	CC1247-CC1267
CC_1263	-2.29	-3.67	-	-		ribosomal protein L6 (rplF)	protein synthesis	ribosomal protein	CC1247-CC1267

CC_1264	-2.12	-3.52	-	-	ribosomal protein L18 (rplR)	protein synthesis	ribosomal protein	CC1247-CC1267
CC_1265	-1.88	-3.03	-	-	ribosomal protein S5 (rpsE)	protein synthesis	ribosomal protein	CC1247-CC1267
CC_1266	-2.00	-3.29	-	-	ribosomal protein L30 (rpmD)	protein synthesis	ribosomal protein	CC1247-CC1267
CC_1267	-1.49	-2.55	-	-	ribosomal protein L15 (rplO)	protein synthesis	ribosomal protein	CC1247-CC1267
CC_1270	-	-1.94	-	-	ribosomal protein S13 (rpsM)	protein synthesis	ribosomal protein	CC1270-CC1272
CC_1271	-	-1.77	-	-	ribosomal protein S11 (rpsK)	protein synthesis	ribosomal protein	CC1270-CC1272
CC_1273	-	-2.07	-	-	ribosomal protein L17 (rplQ)	protein synthesis	ribosomal protein	
CC_1341	-	-1.48	-	-	glycyl-tRNA synthetase, alpha subunit (glyQ) [6.1.1.14]	protein synthesis	tRNA aminoacylation	CC1341-CC1342
CC_1376	-	-2.86	-	-	ribosomal protein L13 (rplM)	protein synthesis	ribosomal protein	CC1376-CC1377
CC_1377	-1.22	-2.89	-	-	ribosomal protein S9 (rpsI)	protein synthesis	ribosomal protein	CC1376-CC1377
CC_1587	-	-1.44	-	-	S-adenosylmethionine tRNA ribosyltransferase-isomerase (queA) {2.4.99.17}	protein synthesis	tRNAmodification	CC1587-CC1588
CC_1667	-1.22	-2.25	-1.71	-1.46	ribosomal protein L9 (rplI)	protein synthesis	ribosomal protein	CC1667-CC1669
CC_1668	-1.20	-	-	-	ribosomal protein S18 (rpsR)	protein synthesis	ribosomal protein	CC1667-CC1669
CC_1669	-1.30	-3.00	-	-	ribosomal protein S6 (rpsF)	protein synthesis	ribosomal protein	CC1667-CC1669
CC_1721	-	-1.60	-	-	ribosomal protein L32 (rpmF)	protein synthesis	ribosomal protein	
CC_1874	-	-1.39	-	-	peptide chain release factor 2 (prfB)	protein synthesis	translation factors	
CC_1892	-	-1.61	-	-	aspartyl-tRNA synthetase (aspS) [6.1.1.12]	protein synthesis	tRNA aminoacylation	
CC_1922	-	-	-1.70	-1.41	translation elongation factor EF-Ts (tsf)	protein synthesis	translation factors	
CC_1923	-	-2.58	-	-	ribosomal protein S2 (rpsB)	protein synthesis	ribosomal protein	CC1922-CC1923
CC_2343	-	-1.31	-	-	translation initiation factor IF-1	protein synthesis	translation factors	CC2340--CC2346
CC_2459	-	-1.83	-	-	ribosomal protein L33 (rpmG)	protein synthesis	ribosomal protein	
CC_2511	-	-2.19	-	-	ribosomal protein S4 (rpsD)	protein synthesis	ribosomal protein	
CC_2529	-	-1.41	-	-	alanyl-tRNA synthetase (alaS) [6.1.1.7]	protein synthesis	tRNA aminoacylation	
CC_3199	-	-	-1.31	-1.21	translation elongation factor EF-Tu (tufA)	protein synthesis	translation factors	
CC_3200	-	-2.00	-1.38	-1.21	translation elongation factor G (fusA)	protein synthesis	translation factors	CC3199-CC3202
CC_3201	-	-2.16	-	-	ribosomal protein S7 (rpsG)	protein synthesis	ribosomal protein	CC3199-CC3202
CC_3202	-1.36	-2.33	-	-	ribosomal protein S12 (rpsL)	protein synthesis	ribosomal protein	CC3199-CC3202
CC_3275	-	-1.95	-	-	ribosomal protein L31 (rpmE)	protein synthesis	ribosomal protein	
CC_3297	-1.23	-1.88	-	-	ribosomal protein S21 (rpsU)	protein synthesis	ribosomal protein	
CC_3321	-	-1.43	-	-	ribosomal protein L36 (rpmJ)	protein synthesis	ribosomal protein	CC3320-CC3321
CC_3587	-1.16	-2.32	-1.47	-1.82	ribosomal protein S1 (rpsA)	protein synthesis	ribosomal protein	
CC_3651	-	-1.83	-	-	16S rRNA processing protein RimM (rimM)	protein synthesis	ribosome biogenesis	
CC_3652	-1.66	-3.34	-	-	ribosomal protein S16 (rpsP)	protein synthesis	ribosomal protein	CC3651-CC3653

CC_1572	-	1.58	-	-	arginyl-tRNA-protein transferase (2.3.2.8)	protein fate	protein modification	
CC_1986	1.55	1.66	-	-	prolyl oligopeptidase family protein	protein fate	degradation of proteins, peptides, and glycopeptides	
CC_2104	-	1.47	-	-	peptidase C13 family	protein fate	degradation of proteins, peptides, and glycopeptides	CC2102-CC2104
CC_2468	-	-	2.97	2.22	ATP-dependent Clp protease, ATP-binding subunit ClpA (clpA)	protein fate	degradation of proteins, peptides, and glycopeptides	
CC_2539	-	-	1.01	-	M61 glycyl aminopeptidase	protein fate	degradation of proteins, peptides, and glycopeptides	
CC_2746	1.37	1.70	-	-	membrane dipeptidase	protein fate	degradation of proteins, peptides, and glycopeptides	
CC_2840	1.26	-	-	-	peptidase family M28	protein fate	degradation of proteins, peptides, and glycopeptides	
CC_3287	-	2.14	-	-	M61 glycyl aminopeptidase	protein fate	degradation of proteins, peptides, and glycopeptides	
CC_3584	-	-	1.81	2.09	peptidase, M16 family	protein fate	degradation of proteins, peptides, and glycopeptides	
CC_3672	-	1.70	-	-	heme exporter protein CcmC (ccmC)	protein fate	cytochrome synthesis	CC3671-CC3673
CC_3702	-	1.40	-	-	peptidyl-dipeptidase Dcp (dcp) [3.4.15.5]	protein fate	degradation of proteins, peptides, and glycopeptides	
CC_0010	-1.27	-1.61	-1.25	-1.06	dnaK protein (dnaK)	protein fate	protein folding and stabilization	CC0010-CC0011
CC_0011	-1.70	-2.19	-	-	dnaJ protein (dnaJ)	protein fate	protein folding and stabilization	CC0010-CC0011
CC_0685	-2.22	-2.68	-1.84	-1.79	chaperonin, 60 kDa (groEL)	protein fate	protein folding and stabilization	CC0685-CC0686
CC_0686	-2.21	-2.82	-	-	chaperonin, 10 kDa (groES)	protein fate	protein folding and stabilization	CC0685-CC0686
CC_1268	-1.26	-1.88	-	-	preprotein translocase, SecY subunit (secY)	protein fate	protein and peptide secretion and trafficking	CC1268-CC1269
CC_1476	-1.34	-1.59	-	-	heat shock protein, Hsp70 family	protein fate	protein folding and stabilization	
CC_1582	-	-1.35	-	-	peptidyl-prolyl cis-trans isomerase, cyclophilin-type	protein fate	protein folding and stabilization	CC1582-CC1584
CC_1885	-	-1.47	-	-	leucyl/phenylalanyl-tRNA--protein transferase (aat) [2.3.2.6]	protein fate	protein modification	CC1883-CC1885
CC_1964	-	-1.69	-1.52	-1.40	trigger factor (tig) [5.2.1.8]	protein fate	protein folding and stabilization	
CC_1991	-	-1.25	-	-	protein-export membrane protein SecD (secD)	protein fate	protein and peptide secretion and trafficking	CC1989-CC1992
CC_1992	-	-1.37	-	-	preprotein translocase, YajC subunit (yajC)	protein fate	protein and peptide secretion and trafficking	CC1989-CC1992
CC_2258	-	-1.62	-	-	heat shock protein, Hsp20 family	protein fate	protein folding and stabilization	
CC_3679	-1.40	-1.68	-	-	signal recognition particle-docking protein FtsY (ftsY)	protein fate	protein and peptide secretion and trafficking	

CC_3731	-	1.31	-	-	endonuclease III (nth) [4.2.99.18]	DNA and RNA metabolism	DNA replication, recombination, and repair	
CC_0034	-	-1.41	-1.46	-1.65	polyribonucleotide nucleotidyltransferase (pnp) [2.7.7.8]	DNA and RNA metabolism	RNA degradation	
CC_0044	-	-	-	-1.55	N utilization substance protein A (nusA)	DNA and RNA metabolism	transcription factors	
CC_0502	-	-1.54	-	-1.55	DNA-directed RNA polymerase, beta subunit (rpoB) [2.7.7.6]	DNA and RNA metabolism	RNA synthesis	CC0502-CC0503

CC_0503	-	-1.51	-	-		DNA-directed RNA polymerase, beta subunit (rpoC) [2.7.7.6]	DNA and RNA metabolism	RNA synthesis	CC0502-CC0503
CC_0835	-1.55	-2.20	-	-		ATP-dependent RNA helicase, DEAD/DEAH box family	DNA and RNA metabolism	RNA unwinding	
CC_1272	-	-1.84	-1.09	-		DNA-directed RNA polymerase, alpha subunit (rpoA) [2.7.7.6]	DNA and RNA metabolism	RNA synthesis	CC1270-CC1272
CC_1580	-	-	-1.21	-1.43		DNA gyrase subunit A (gyrA) [5.99.1.3]	DNA and RNA metabolism	DNA replication, recombination, and repair	
CC_1665	-1.14	-	-	-		replicative DNA helicase (dnaB) [3.6.4.12]	DNA and RNA metabolism	DNA replication, recombination, and repair	
CC_1847	-	-1.44	-	-		ATP-dependent RNA helicase, DEAD/DEAH box family	DNA and RNA metabolism	RNA unwinding	
CC_1877	-	-1.41	-	-		ribonuclease, Rne/Rng family protein {3.1.26.12}	DNA and RNA metabolism	RNA degradation	
CC_3205	-	-1.69	-	-		transcription termination/antitermination factor NusG (nusG)	DNA and RNA metabolism	transcription factors	
CC_3760	-1.52	-2.15	-	-		transcription termination factor Rho (rho)	DNA and RNA metabolism	transcription factors	

CC_0081	-	1.45	-	-		transcriptional regulator, MerR family	regulatory functions	gene expression regulation	
CC_0284	-	2.23	-	-	SigT/SigU	response regulator LovR	regulatory functions	gene expression regulation	CC0284-CC0285
CC_1649	-	1.62	-	-		RNA polymerase sigma-70 factor, ECF subfamily	regulatory functions	sigma factor	CC1649-CC1650
CC_2576	1.31	2.11	-	-		response regulator	regulatory functions	gene expression regulation	
CC_2883	2.26	2.68	-	-	SigT/SigU	RNA polymerase sigma-70 factor, ECF subfamily	regulatory functions	sigma factor	
CC_3475	1.20	1.67	-	-	SigT	RNA polymerase sigma-70 factor, ECF subfamily	regulatory functions	sigma factor	CC3473-CC3475
CC_3476	-	1.23	-	-	SigT	anti-sigma factor NepR	regulatory functions	gene expression regulation	
CC_3477	-	1.26	1.81	3.21	SigT	response regulator PhyR	regulatory functions	gene expression regulation	
CC_3596	-	1.82	-	-		PTS IIA-like nitrogen-regulatory protein PtsN (ptsN)	regulatory functions	unknown function	CC3596-CC3597
CC_0294	-1.73	-1.64	-	-		phosphate regulon response regulator PhoB	regulatory functions	gene expression regulation	CC0290-CC0294
CC_1339	-	-1.81	-	-		nitrogen regulatory protein P-II 2 (glnK)	regulatory functions	sensory protein	
CC_1776	-2.99	-	-	-		transcriptional regulator, GntR family	regulatory functions	gene expression regulation	
CC_1968	-1.45	-2.04	-	-		nitrogen regulatory protein, P-II family	regulatory functions	sensory protein	CC1968-CC1969
CC_2527	-	-1.11	-	-		transcriptional regulator, AraC family	regulatory functions	gene expression regulation	
CC_3047	-	-1.27	-	-		RNA polymerase sigma factor RpoD (rpoD)	regulatory functions	sigma factor	
CC_3623	-1.12	-	-	-		sensor histidine kinase/response regulator	regulatory functions	gene expression regulation	

CC_0074	-	1.25	-	-		hypothetical protein	protein of unknown function	unknown function	CC0074-CC0075
CC_0125	-	1.71	-	-		hypothetical protein	protein of unknown function	unknown function	CC0125-CC0127
CC_0127	-	1.43	-	-		MAPEG family protein	protein of unknown function	enzymes of unknown specificity	CC0125-CC0127
CC_0128	-	1.40	-	-	SigT	KTSC protein	protein of unknown function	unknown function	
CC_0280	2.05	2.36	-	-	SigT/SigU	hypothetical protein	protein of unknown function	unknown function	
CC_0501	-	1.77	-	-	SigT/SigU	hypothetical protein	protein of unknown function	unknown function	
CC_0512	-	1.28	-	-		hypothetical protein	protein of unknown function	unknown function	
CC_0550	-	1.95	-	-		hypothetical protein	protein of unknown function	unknown function	
CC_0554	1.64	2.10	-	-		hypothetical protein	protein of unknown function	unknown function	
CC_0555	1.29	-	-	-		FAD binding protein	protein of unknown function	unknown function	CC0555-CC0557
CC_0556	1.25	1.86	-	-	SigT	MoxR-like ATPase	protein of unknown function	unknown function	CC0555-CC0557
CC_0558	1.68	1.94	-	-		hypothetical protein	protein of unknown function	unknown function	
CC_0559	1.43	2.02	-	-		hypothetical protein	protein of unknown function	unknown function	
CC_0673	2.18	-	-	-		DUF1328 containing protein	protein of unknown function	unknown function	
CC_0681	2.00	1.99	-	-		hypothetical protein	protein of unknown function	unknown function	
CC_0682	3.11	3.83	-	-		hypothetical protein	protein of unknown function	unknown function	
CC_0697	-	1.58	-	-		TraB domaing containig protein	protein of unknown function	unknown function	CC0696-CC0697
CC_0698	-	1.80	-	-		TraB domaing containig protein	protein of unknown function	unknown function	
CC_0781	-	1.85	-	-		hypothetical protein	protein of unknown function	unknown function	
CC_0956	2.91	3.15	-	-	SigT/SigU	hypothetical protein	protein of unknown function	unknown function	
CC_1115	-	-	1.28	1.89		hypothetical protein	protein of unknown function	unknown function	
CC_1179	2.71	2.78	-	-	SigT/SigU	hypothetical protein	protein of unknown function	unknown function	CC1178-CC1179
CC_1453	2.38	2.54	-	-	SigT	hypothetical protein	protein of unknown function	unknown function	CC1452-CC1453
CC_1532	4.76	4.13	-	-	SigT/SigU	DUF2171 domain containing protein	protein of unknown function	unknown function	
CC_1629	3.22	2.11	3.32	-		xylose isomerase/endonuclease family protein	protein of unknown function	enzymes of unknown specificity	CC1628-CC1632
CC_1631	3.29	-	-	-		xylose isomerase/endonuclease family protein	protein of unknown function	enzymes of unknown specificity	CC1628-CC1632
CC_1633	3.50	2.36	-	-		DUF1080 containing protein	protein of unknown function	unknown function	
CC_1636	1.68	1.59	-	-		DUF2147 containing protein	protein of unknown function	unknown function	
CC_1650	-	1.28	-	-		hypothetical protein	protein of unknown function	unknown function	CC1649-CC1650
CC_1713	-	1.64	-	-		hypothetical protein	protein of unknown function	unknown function	
CC_1848	-	2.83	-	-		DUF563 containing protein	protein of unknown function	unknown function	
CC_2026	-	1.34	-	-		hypothetical protein	protein of unknown function	unknown function	
CC_2030	-	1.84	-	-		hypothetical protein	protein of unknown function	unknown function	CC2030-CC2031
CC_2031	1.29	2.08	-	-		TPR domain protein	protein of unknown function	unknown function	CC2030-CC2031
CC_2067	1.45	2.21	-	-	SigT	hypothetical protein	protein of unknown function	unknown function	
CC_2111	-	1.97	-	-		DUF307 containing protein	protein of unknown function	unknown function	

CC_2122	-	1.41	2.11	1.96		DUF924 containing protein	protein of unknown function	unknown function	
CC_2348	3.98	5.29	-	-		hypothetical protein	protein of unknown function	unknown function	
CC_2362	-	-	1.22	1.25		PspA/IM30 family protein	protein of unknown function	unknown function	
CC_2365	1.27	1.56	-	-		DUF2491 domain containing protein	protein of unknown function	unknown function	
CC_2435	1.95	3.72	-	-		DUF3315 containing protein	protein of unknown function	unknown function	
CC_2549	2.87	3.06	-	-	SigT	SOUL heme-binding protein	protein of unknown function	unknown function	
CC_2589	-	1.25	-	-		hypothetical protein	protein of unknown function	unknown function	
CC_2626	2.03	3.08	-	-		CBS domain protein	protein of unknown function	unknown function	CC2626-CC2627
CC_2645	1.65	2.78	-	-		hypothetical protein	protein of unknown function	unknown function	
CC_2676	-	1.77	-	-		hypothetical protein	protein of unknown function	unknown function	
CC_2678	-	1.52	-	-		hypothetical protein	protein of unknown function	unknown function	
CC_2745	1.91	2.23	-	-		DUF2272 containing protein	protein of unknown function	unknown function	
CC_2823	-	-	-	1.71		TldD/PmbA family protein	protein of unknown function	unknown function	
CC_2914	-	1.56	-	-		hypothetical protein	protein of unknown function	unknown function	CC2914-CC2915
CC_2964	-	1.45	-	-		DUF1003 containing protein	protein of unknown function	unknown function	
CC_2965	1.47	1.85	-	-		DUF1508 containing protein	protein of unknown function	unknown function	
CC_3178	-	1.57	-	-		pirin-related protein	protein of unknown function	unknown function	
CC_3207	1.90	2.95	-	-		hypothetical protein	protein of unknown function	unknown function	
CC_3260	1.84	2.51	-	-	SigT	TspO/MBR family	protein of unknown function	unknown function	
CC_3265	-	1.24	-	-		hypothetical protein	protein of unknown function	unknown function	
CC_3291	4.55	4.96	-	-		DUF465 containing protein	protein of unknown function	unknown function	
CC_3294	-	1.63	-	-		DUF1192) containing protein	protein of unknown function	unknown function	
CC_3335	3.33	4.34	-	-		hypothetical protein	protein of unknown function	unknown function	
CC_3374	1.33	1.31	-	-		NfeD-like C-terminal, partner-binding	protein of unknown function	unknown function	CC3374-CC3375
CC_3375	-	1.39	-	-		stomatin-like proteins	protein of unknown function	unknown function	CC3374-CC3375
CC_3455	-	1.90	-	-		LemA family	protein of unknown function	unknown function	CC3455-CC3456
CC_3457	-	1.22	-	-		hypothetical protein	protein of unknown function	unknown function	
CC_3466	3.47	3.56	4.57	3.51	SigT/SigU	DUF883 containing protein	protein of unknown function	unknown function	
CC_3546	-	1.93	-	-		Usg protein	protein of unknown function	unknown function	CC3541-CC3546
CC_3710	1.40	1.88	-	-		hypothetical protein	protein of unknown function	unknown function	CC3710-CC3711
CC_3711	1.47	1.81	-	-		hypothetical protein	protein of unknown function	unknown function	CC3710-CC3711
CC_3757	-	1.26	-	-		hypothetical protein	protein of unknown function	unknown function	CC3756-CC3757
CC_0046	-	-1.29	-	-		hypothetical protein	protein of unknown function	unknown function	
CC_0048	-	-1.70	-	-		hypothetical protein	protein of unknown function	unknown function	
CC_0215	-1.05	-1.87	-	-		SapC-related protein	protein of unknown function	unknown function	
CC_0287	-2.82	-2.83	-	-		hypothetical protein	protein of unknown function	unknown function	
CC_0320	-2.00	-1.85	-	-		hypothetical protein	protein of unknown function	unknown function	CC0320-CC0321
CC_0347	-	-1.27	-	-		Integral membrane protein TerC family	protein of unknown function	unknown function	
CC_0359	-1.32	-1.85	-	-		deoxyhypusine synthase family protein	protein of unknown function	unknown function	
CC_0669	-3.84	-4.14	-	-		hypothetical protein	protein of unknown function	unknown function	
CC_0844	-1.22	-1.58	-	-		hypothetical protein	protein of unknown function	unknown function	
CC_1042	-	-1.41	-	-		hypothetical protein	protein of unknown function	unknown function	
CC_1120	-3.36	-4.12	-	-		DUF934 containing protein	protein of unknown function	unknown function	CC1119-CC1121
CC_1246	-1.60	-3.03	-	-		hypothetical protein	protein of unknown function	unknown function	
CC_1472	-1.56	-1.36	-	-		hypothetical protein	protein of unknown function	unknown function	
CC_1494	-1.07	-1.71	-	-		hypothetical protein	protein of unknown function	unknown function	
CC_1728	-	-1.96	-	-		hypothetical protein	protein of unknown function	unknown function	
CC_1775	-2.45	-2.07	-	-		DUF2946 containingl protein	protein of unknown function	unknown function	
CC_1780	-3.77	-	-	-		ThiJ/PfpI family protein	protein of unknown function	unknown function	
CC_1783	-1.56	-1.65	-	-		hypothetical protein	protein of unknown function	unknown function	
CC_1943	-	-1.48	-	-		hypothetical protein	protein of unknown function	unknown function	CC1933--CC1952
CC_1951	-	-1.03	-	-		SnoaL-like domain	protein of unknown function	unknown function	CC1933-CC1952
CC_2160	-2.56	-	-	-		phasin family protein	protein of unknown function	unknown function	
CC_2370	-	-1.81	-	-		membrane protein, putative	protein of unknown function	unknown function	
CC_2506	-	-1.22	-	-		thioesterase-like superfamily	protein of unknown function	unknown function	CC2505-CC2506
CC_3117	-	-1.22	-	-		hypothetical protein	protein of unknown function	unknown function	CC3117-CC3118
CC_3124	-1.39	-	-	-		hypothetical protein	protein of unknown function	unknown function	
CC_3319	-	-1.94	-	-		DUF2312 containig protein	protein of unknown function	unknown function	
CC_3446	-	-1.91	-	-		hypothetical protein	protein of unknown function	unknown function	CC3445-CC3450
CC_3499	-3.15	-3.42	-	-		hypothetical protein	protein of unknown function	unknown function	CC3499-CC3500
CC_3591	-	-1.79	-	-		hypothetical protein	protein of unknown function	unknown function	
CC_3613	-	-1.30	-	-		hypothetical protein	protein of unknown function	unknown function	

[a] according to CMR ("Comprehensive Microbial Resource") annotation.

[b] DNA microarray analyses. The ratios were determined by microarray hybridization of RNA samples isolated from exponential phase cells immediately before or after expourse to either 110 mM sucrose (suc) or 60 mM NaCl for 30 min. Values are the log2 ratio as mentioned. Genes displaying ratio of < -1.00 or > 1.00 were assumed as differentially expressed and values are highlighted in red (upregulated) or green (downregulated). Results shown are the average of three independent biological experiments.

[c] 2D proteome analyses. Cultures of parental cells NA1000 were grown to the exponential growth phase and submitted for 30 minutes to either 110 mM sucrose (suc) or 60 mM NaCl for 30 min or kept under no stress conditions. Values are the log2 ratio as mentioned and correspond to the relative intensity of the same spot in each sample analyzed. Proteins were identified by mass spectrometry. Results shown are the average of two independent biological experiments.

[d] genes found to be upregulated (red) or downregulated (green) in a previous transcriptomic and proteomic analysis of parental NA1000 cells upon carbon starvation.

[e] genes whose putative transcriptional regulator(s) is (are) known.

[f] according to a reanalysis of the deduced protein sequences by using Pfam and SMART tools to search for conserved domains. In addition, the sequences were compared with proteins available in database using Blastp.

[g] problabe cellular process in which the corresponding gene is involved. Genes and proteins were clustered according to the reanalysis of the deduced protein sequences.

[h] the operons were predicted based on genome localization and the signal from the different probes used in transcriptome analyses.
