# Supplementary material for: A comprehensive genomic, transcriptomic and proteomic analysis of a hyperosmotic stress sensitive α-proteobacterium
Source: BMC Microbiol. 2015 Mar 26;15:71. doi: 10.1186/s12866-015-0404-x (PMC4391529; doi:10.1186/s12866-015-0404-x)
Supplement: Additional file 3: Figure S1. — Proteomic analysis of stresses cells. Panels display false colored dual channel images of the synthesis of proteins after 30 min as revealed by radioactive labeling for 5 min. All significantly regulated proteins are labeled in their appropriate color (green or red). Delta 2D software was used to visualize complex protein expression patterns on the 2D image in the standard pH range of 3 to 10. [file 12866_2015_404_MOESM3_ESM.pdf]

sucrose x no stress

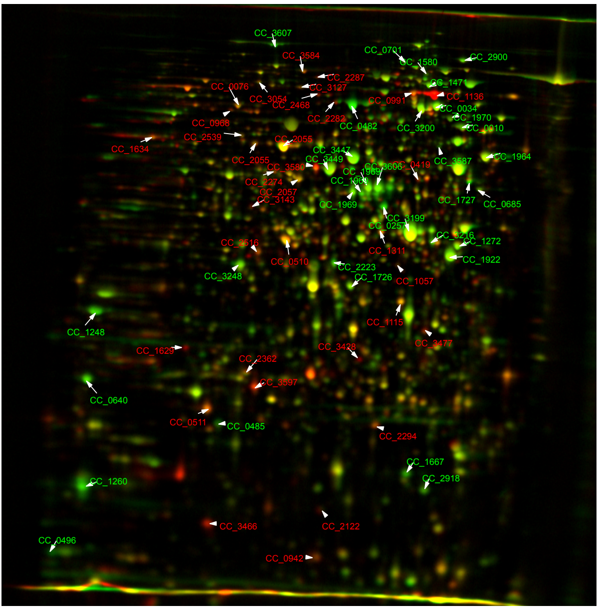

NaCl x no stress

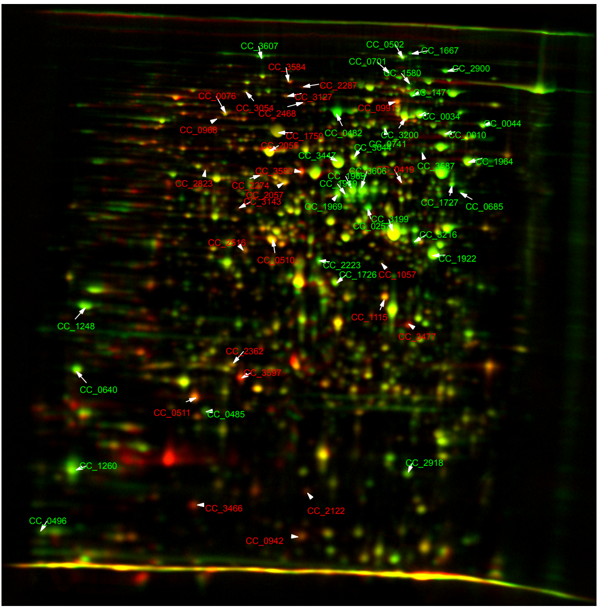

sucrose x NaCl

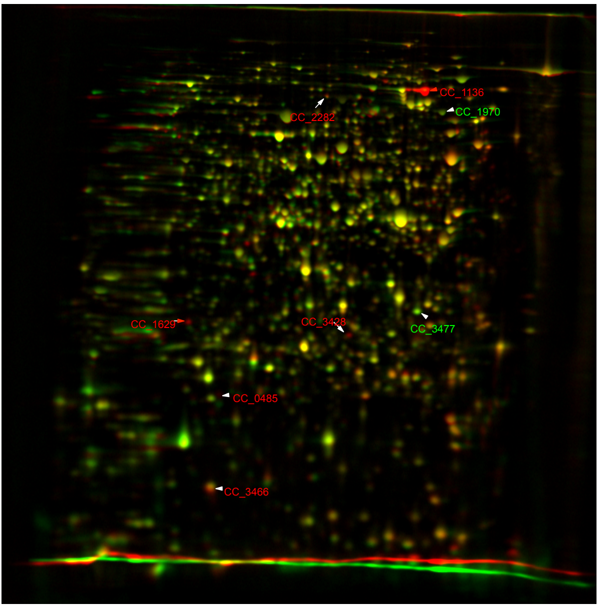

Figure S1. Proteomic analysis of stressed cells. Panels display false colored dual channel images of the synthesis of proteins after 30 min as revealed by radioactive labeling for 5 min. All significantly regulated proteins are labeled in their appropriate color (green or red). Delta 2D software was used to visualize complex protein expression patterns on the 2D image in the standard pH range of 3 to 10.
