# Supplementary material for: A comprehensive genomic, transcriptomic and proteomic analysis of a hyperosmotic stress sensitive α-proteobacterium
Source: BMC Microbiol. 2015 Mar 26;15:71. doi: 10.1186/s12866-015-0404-x (PMC4391529; doi:10.1186/s12866-015-0404-x)
Supplement: Additional file 4: Figure S2. — TIGRFam Treemaps of regulated C. crescentus genes (circles) and proteins (cells) after osmotic stress found by global transcriptomic and proteomic analyses. (A) Treemap for the response to sucrose stress, (B) Treemap for the response to NaCl stress and (C) Treemap showing the comparison between sucrose and NaCl stress. Figures on left side show the results on the level of functional category/subcategory and on the right side on the level of genes and proteins. Blue colors show repressed genes and orange colors the induction of genes. The intensity of the colors reflects the strength of repression or induction. [file 12866_2015_404_MOESM4_ESM.pdf]

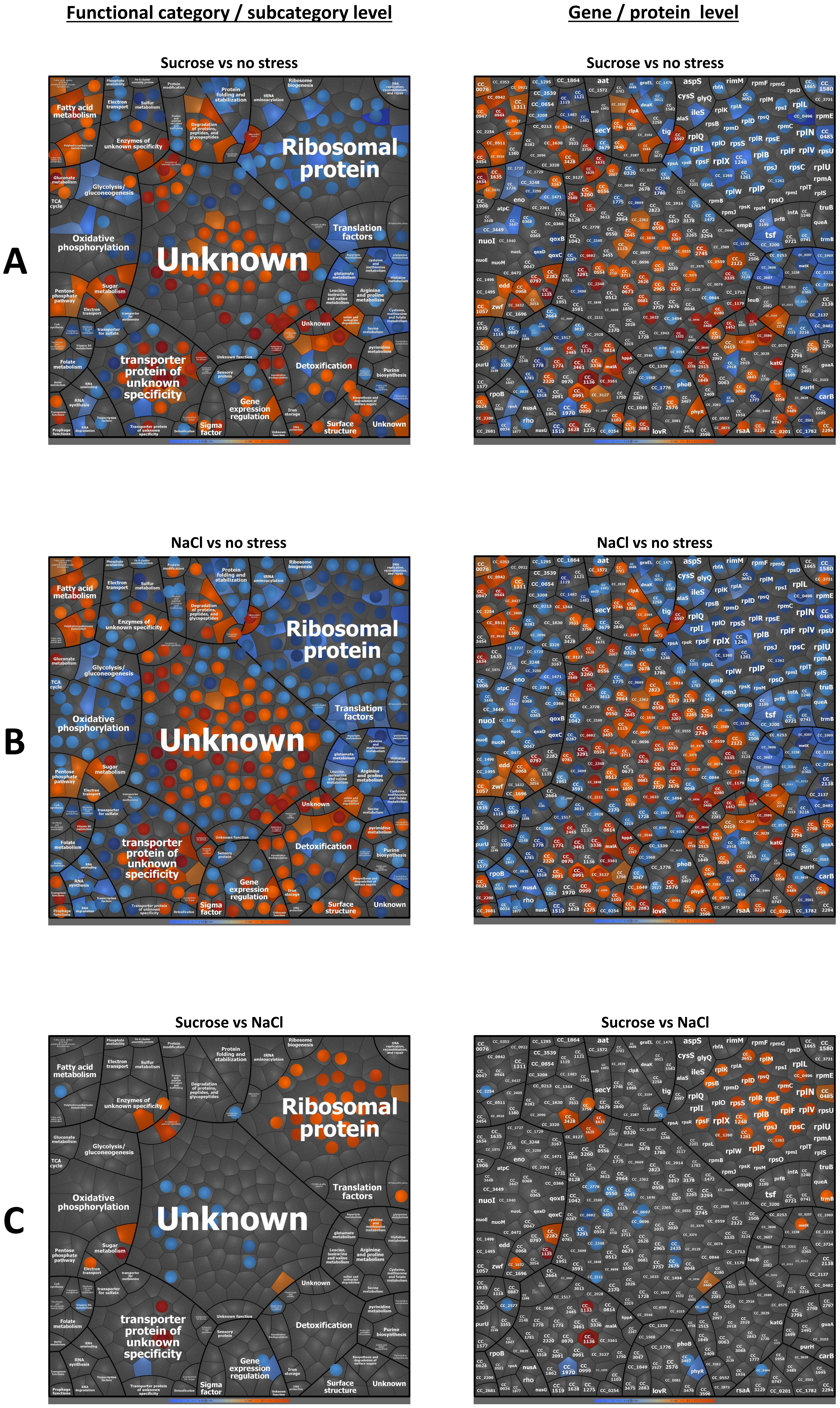

Figure S2. TIGRFam Treemaps of regulated *C. crescentus* genes (circles) and proteins (cells) after osmotic stress found by global transcriptomic and proteomic analyses. (**A**) Treemap for the response to sucrose stress, (**B**) Treemap for the response to NaCl stress and (**C**) Treemap showing the comparison between sucrose and NaCl stress. Figures on left side show the results on the level of functional category / subcategory and on the right side on the level of genes and proteins. Blue colors show repressed genes and orange colors the induction of genes. The intensity of the colors reflects the strength of repression or induction.
